# Tribody [(HER2)_2_xCD16] Is More Effective Than Trastuzumab in Enhancing γδ T Cell and Natural Killer Cell Cytotoxicity Against HER2-Expressing Cancer Cells

**DOI:** 10.3389/fimmu.2018.00814

**Published:** 2018-04-19

**Authors:** Hans H. Oberg, Christian Kellner, Daniel Gonnermann, Susanne Sebens, Dirk Bauerschlag, Martin Gramatzki, Dieter Kabelitz, Matthias Peipp, Daniela Wesch

**Affiliations:** ^1^Institute of Immunology, University Hospital Schleswig-Holstein (UKSH), Christian-Albrechts University (CAU) of Kiel, Kiel, Germany; ^2^Division of Stem Cell Transplantation and Immunotherapy, Department of Medicine II, University Hospital Schleswig-Holstein (UKSH), Christian-Albrechts University (CAU) of Kiel, Kiel, Germany; ^3^Institute for Experimental Cancer Research, University Hospital Schleswig-Holstein (UKSH), Christian-Albrechts University (CAU) of Kiel, Kiel, Germany; ^4^Clinic of Gynecology and Obstetrics, University Hospital Schleswig-Holstein (UKSH), Christian-Albrechts University (CAU) of Kiel, Kiel, Germany

**Keywords:** human γδ T cells, human natural killer cells, human epidermal growth factor receptor 2, trastuzumab, bispecific antibody, tribody, cancer, CD16

## Abstract

An enhanced expression of human epidermal growth factor receptor 2 (HER2, ErbB2) often occurs in an advanced stage of breast, ovarian, gastric or esophageal cancer, and pancreatic ductal adenocarcinoma (PDAC). Commonly, HER2 expression is associated with poor clinical outcome or chemoresistance in ovarian and breast cancer patients. Treatment with humanized anti-HER2 monoclonal antibodies, such as trastuzumab or pertuzumab, has improved the outcome of patients with HER2-positive metastatic gastric or breast cancer, but not all patients benefit. In this study, the bispecific antibody [(HER2)_2_xCD16] in the tribody format was employed to re-direct CD16-expressing γδ T lymphocytes as well as natural killer (NK) cells to the tumor-associated cell surface antigen HER2 to enhance their cytotoxic anti-tumor activity. Tribody [(HER2)_2_xCD16] comprises two HER2-specific single chain fragment variable fused to a fragment antigen binding directed to the CD16 (FcγRIII) antigen expressed on γδ T cells and NK cells. Our results revealed the superiority of tribody [(HER2)_2_xCD16] compared to trastuzumab in triggering γδ T cell and NK cell-mediated lysis of HER2-expressing tumor cells, such as PDAC, breast cancer, and autologous primary ovarian tumors. The increased efficacy of [(HER2)_2_xCD16] can be explained by an enhanced degranulation of immune cells. Although CD16 expression was decreased on γδ T cells in several PDAC patients and the number of tumor-infiltrating NK cells and γδ T cells was impaired in ovarian cancer patients, [(HER2)_2_xCD16] selectively enhanced cytotoxicity of cells from these patients. Here, unique anti-tumor properties of tribody [(HER2)_2_xCD16] are identified which beyond addressing HER2 overexpressing solid tumors may allow to treat with similar immunoconstructs combined with the adoptive transfer of γδ T cells and NK cells refractory hematological malignancies. A major advantage of γδ T cells and NK cells in the transplant situation of refractory hematological malignancies is given by their HLA-independent killing and a reduced graft-*versus*-host disease.

## Introduction

The human epidermal growth factor receptor 2 [(HER2), ErbB2] stimulates tumor cell proliferation *via* the Ras-MAP-kinase pathway and its expression is often associated with an aggressive tumor phenotype, advanced stage diseases, and poor clinical outcome (1, 2). Since anti-HER2 therapies are successful for the treatment of HER2-expressing tumors, HER2 is often selected as a tumor target antigen (3). HER2 expression in cardiomyocytes does not exclude an anti-HER2 therapy when the cardiac function in patients receiving anti-HER2 therapy is closely monitored. A dysfunction of cardiomyocytes, which is induced in 4% of the cancer patients receiving anti-HER2 therapy, is reversible (4). However, several HER2-positive tumors are resistant against anti-HER2 therapy or develop a resistance often accompanied by loss of anti-HER2-directed Th1 immunity (5). In an attempt to optimize anti-HER2 therapies, the initial monotherapy with humanized anti-HER2 mAb trastuzumab (Herceptin^®^, Genentech, South San Francisco, CA, USA) against metastatic gastric or breast cancer was gradually replaced by combination therapies with cytostatic agents (e.g., docetaxel, capecitabine, paclitaxel) and/or other anti-HER2 mAb (e.g., pertuzumab), and/or tyrosine kinase inhibitors (e.g., lapatinib) (2, 3, 6–12). Alternatively, the antibody-drug conjugate (ADC) trastuzumab emtansine (T-DM-1) consisting of the anti-HER2 mAb trastuzumab linked to the cytotoxic agent emtansine (DM-1), which enters and destroys the HER2-overexpressing cells by binding to tubulin, was successful in patients with advanced breast cancer (13, 14). Trastuzumab and pertuzumab induce antibody-dependent cell-mediated cytotoxicity (ADCC) and/or cell death of tumor cells by inhibition of HER2 signaling (15–17). ADCC is mediated by activating Fcγ-receptor (FcγR) bearing myeloid cells as well as by natural killer (NK) cells or γδ T lymphocytes (10, 18–20). Regarding γδ T cells, Capietto and colleagues recently reported that adoptive transfer of human Vγ9Vδ2-expressing γδ T lymphocytes from healthy donors (HDs) together with trastuzumab reduced growth of HER2-expressing breast cancer tumors grafted into immunocompromised mice. In their study, γδ T cells bound to mAb-labeled breast cancer tumors *via* FcγRIII (CD16) and thereby exerted ADCC (21).

Differential clinical responses toward therapeutic antibodies such as trastuzumab or rituximab related to polymorphisms in *FCGRIIA* and *FCGRIIIA* genes have promoted the development of Fc engineered antibodies, which improve cellular cytotoxicity against tumors (16, 17, 22, 23). Besides, enhanced cytotoxicity was also obtained with bispecific antibodies (bsAb), which allow redirecting of distinct effector cell populations including T lymphocytes to the tumor-site (24). The development of bsAb recruiting T cells has been successfully introduced into clinical application for blinatumomab and catumaxomab for treatment of relapsed or refractory B-cell precursor acute lymphoblastic leukemia and malignant ascites, respectively (25–27). Targeting solid tumors with bsAb is more complex and is under investigation (28, 29). bsAb also offer the ability to selectively trigger a distinct activating FcγR with high affinity and allow recruitment of FcγR expressing effector cells. For example, bispecific [HER2xCD16] antibodies retargeting NK cells to tumors have proven efficacy (30–32).

γδ T lymphocytes are attractive effector cells for bsAb based on their HLA-independent recognition of antigens, their capacity to present antigens to αβ T cells and not to induce a severe graft-*versus*-host disease (33–36). Previously, we have shown the efficacy of bsAb, such as [HER2xCD3] and [(HER2)_2_xVγ9], which selectively target CD3- and/or Vγ9-expressing T cells to HER2-expressing pancreatic ductal adenocarcinoma (PDAC) cells. HER2 is overexpressed on tumor cells of patients with PDAC (37). In particular, [(HER2)_2_xVγ9] selectively enhanced the cytotoxicity of Vγ9-expressing T cells from PDAC patients *in vitro* by inducing the release of perforin and granzyme B as well as *in vivo* in xenograft models of PDAC upon transfer of human γδ T cells (38). These data are of great interest because PDAC is an extremely aggressive disease with poor prognosis and limited therapeutic options due to the resistance of PDAC to chemotherapy (39). Similar to PDAC, ovarian cancer (OC) is also regarded as an aggressive malignancy with a characteristic immunosuppressive tumor microenvironment (40). Ultimate recurrence of (serous) OC after surgery is often associated with poor prognosis and resistance against chemotherapy (41). In this study, we focused on a bsAb in the tribody format, [(HER2)_2_xCD16], to redirect CD16-expressing γδ T cells in addition to NK cells to lyse HER2-expressing tumor cells. For this purpose, γδ T cells and NK cells were used for cytotoxity assays, and applied either as peripheral blood lymphocytes (PBL) or tumor-infiltrating lymphocytes (TIL), as purified cells isolated out of PBL or TIL (freshly isolated) or as short-term *in vitro* expanded cells (suitable for adoptive cell transfer). The potential of tribody [(HER2)_2_xCD16] to enhance the cytotoxicity of these cells against allogeneic cancer cell lines and autologous primary OC cells was compared to the cytotoxic activity mediated by trastuzumab.

## Materials and Methods

### Patient Cohorts

Leukocyte concentrates from healthy adult blood donors were obtained from the Department of Transfusion Medicine of the University Hospital Schleswig-Holstein (UKSH) in Kiel, Germany. Heparinized blood was drawn from healthy donors (HDs) of the Institute of Immunology [UKSH, Christian-Albrechts University (CAU)], whereas blood from patients was obtained from the Department of General and Thoracic Surgery or the Clinic of Gynecology and Obstetrics, both of the UKSH in Kiel. In accordance with the Declaration of Helsinki, written informed consent was obtained from all donors, and the research was approved by the relevant institutional review boards (ethic committee of the Medical Faculty of the CAU to Kiel, code number: D405/10, D403/14, D404/14). Twenty-four patients with histologically verified PDAC (10 females, age 67.2 ± 12.1 years; 14 males, age 66.6 ± 6.9 years, stage pT1-4, pN0-1, L0-1, V0-1Pn 0-1, R0-1) were enrolled. None of the patients had been treated with chemo- or radio-therapy prior to this investigation. Moreover, 20 patients (7 females, age 72.3 ± 6.5 years; 13 males, age 67.2 ± 7.5 years) with other (cancer) diseases were included as follows: 3x papillary carcinoma, pancreaticobiliary type, 3x (multi-cystic) serous cystadenoma of the pancreas, 3x neuroendocrine papilla carcinoma, 2x cholangiocarcinoma, 2x bile duct carcinoma, 2x gastrointestinal carcinoma, 1x tubular adenocarcinoma with high-grade epithelial dysplasia, 1x lymph nodes in the pancreatic head, 1x jejunum metastasis, 1x hepatocellular carcinoma, 1x B cell Non-Hodgkin lymphoma. For functional assays, one patient with advanced stage of breast cancer (age 55 years, stage IIIB, high grading) and one patient with OC (OC1, age 60, high-grade serous, stage IIIC) at first diagnosis shortly after surgery and before chemotherapy were additionally enrolled (code number: AZ A157/11, AZ B3277/10). From another OC patient (OC11, age 52, high-grade serous, stage IIIC) tumor material was obtained after exploratory laparotomy before chemotherapy and blood before and after neoadjuvant chemotherapy with carboplatin/paclitaxel (4x, q3w) plus bevacizumab administered at the second cycle. The adjuvant therapy was followed by three further cycles with carboplatin/paclitaxel (3x, q3w). For flow cytometric analysis, PBL and TIL of 15 patients with advanced OC [age 56 ± 13 years, FIGO-stage IB-IVB (serous or mucinous carcinomas)] were enrolled (AZ B3277/10).

### Isolation and *In Vitro* Culture of Lymphocyte Populations

Peripheral blood lymphocytes were isolated from the leukocyte concentrates, from heparinized blood, or from EDTA blood by Ficoll–Hypaque (Biochrom, Berlin) density gradient centrifugation. To separate freshly isolated γδ T cells or NK cells, negative selection kits [T cell receptor (TCR) γδ^+^ T Cell Isolation Kit or NK cell Isolation Kit, Miltenyi Biotec, Bergisch Gladbach], according to the manufacturer’s instructions were used.

To establish short-term γδ T cell lines, PBL were cultured in RPMI 1640 supplemented with 2 mM l-glutamine, 25 mM Hepes, 100 U/mL penicillin, 100 µg/mL streptomycin, 10% FCS (complete medium), and stimulated with their selective antigens 300 nM phosphorylated antigen (PAg) Bromohydrin-pyrophoshate [(BrHPP); kindly provided by Innate Pharma, Marseille, France] or 5 µM of nitrogen-containing bisphosphonates (n-BP) zoledronic acid (Novartis, Basel, Switzerland). 50 U/mL rIL-2 (Novartis) was added every 2 days over a culture period of 14–21 days. After 2–3 weeks, most γδ T cell lines had a purity of >95% Vδ2 γδ T cells. γδ T cells with a purity of <98% were labeled with anti-TCRαβ mAb clone IP26 (Biolegend, San Diego, CA, USA) and subjected to magnetic separation in order to deplete remaining αβ T cells. Magnetically depleted γδ T cells as well as γδ T cells of donors of whom we initially received only 1 mL blood with a low percentage of γδ T cells were re-stimulated to ensure large-scale expansion of pure γδ T cell lines as previously described (42, 43).

### Separation of Tumor-Infiltrating Lymphocytes and Primary Tumor Cells

Tumors from patients with advanced OC removed during surgery were dissected in tumor tissues by the pathologist of the UKSH. Tumor tissue was washed in 10 cm dishes with PBS to remove blood debris. Subsequently, the tumor tissue was minced into approximately 1 mm^3^ pieces and digested in 5–10 mL PBS with enzymes A, K, and R of the Tumor Dissociation Kit (Miltenyi Biotec) for 1 h at 37°C. Digested cell suspension was then passed through a 100 µm cell strainer (Falcon, BD Bioscience, Heidelberg), visually controlled by light microscopy and centrifuged at 481 *g* for 5 min. Cell pellet was resuspended in complete medium and cultured in 75 cm^2^ culture flasks to establish primary tumor cell lines. TILs were isolated by Ficoll–Hypaque density gradient centrifugation. Polyploidy of primary tumor cells was analyzed by using the tricolor probe TERC (3q26)/MYC (8q24)/SE 7 TC (Kreatech/Leica, #KBI-10704) for fluorescence *in situ* hybridization (FISH) as previously described (44). Polyploidy was detected in 60–100% of cells in each investigated primary ovarian tumor sample which classified them together with a high expression of HER2 and epithelial cell adhesion molecule as cancer cells.

### Tumor Cell Lines

Pancreatic ductal adenocarcinoma cell lines, such as PancTu-I, Panc1, and Panc89 were kindly provided by Dr. Christian Röder, Institute for Experimental Cancer Research UKSH/CAU, Kiel. PDAC cell lines, Burkitt lymphoma Raji cells (DSMZ, Braunschweig) as well as OC cell line SK-OV-3 or IGROV-1 and esophageal cell line OE33 (ATCC, Manassas, VA, USA) were cultured in complete medium. The genotype of tumor cell lines was recently confirmed by short tandem repeats analysis and mycoplasma negativity routinely once per month by RT-PCR. For removing adherent tumor cells from flasks, cells were treated with 0.05% trypsin/0.02% EDTA.

### Generation of bsAb

The tribodies [(HER2)_2_xCD16] (45), [(CD20)_2_xCD16] (45), and [(HER2)_2_xCD89] (unpublished) were expressed as published previously (45). Briefly, Lenti-X™ 293T-cells were co-transfected with corresponding expression vectors coding for light or heavy chain derivatives of the tribodies using the calcium phosphate technique including 5 mM chloroquine. Heterodimeric tribody molecules composed of a light chain and a heavy chain tagged with a C-terminal hexahistidine motif were purified from supernatant by two successive steps of affinity chromatography using CaptureSelect Fab kappa affinity matrix (BAC B.V., Naarden, The Netherlands) and nickel-nitrilotriacetic acid agarose beads (Qiagen, Hilden, Germany) as described earlier (38, 45). Purity and integrity of bsAb were verified by capillary electrophoresis using an Experion™ Automated Electrophoresis System (Bio-Rad, München).

### Flow Cytometry

Fluorochome-labeled mAb were used for six-color surface staining as follows: anti-CD3 clone SK7, anti-TCRγδ clone 11F2 (both from BD Biosciences), anti-TCRαβ clone IP26 (Biolegend), anti-TCRVδ2 clone Immu389 (Beckman Coulter, Krefeld), anti-TCRVδ1 clone TS8.2 (Thermo Fisher Scientific, Schwerte), and anti-CD56 clone NCAM16.2 (BD Biosciences), and corresponding isotype controls (BD Biosciences or Biolegend). Fluorochome-labeled mAb were used for three-color surface staining as follows: anti-TCRγδ clone 11F2 (BD Biosciences), anti-TCRVδ2 clone Immu389 (Beckman Coulter), and anti-CD16 clone B73.1 (BD Biosciences) or anti-CD3 clone SK7 (BD Biosciences) plus corresponding isotype controls (BD Biosciences or Biolegend). To analyze HER2 expression, we used 20 µg/mL trastuzumab (Roche Pharma AG, Grenzach Wyhlen), and the corresponding humanized IgG control (20 µg/mL) followed by a second step staining with goat-anti-human *F*(ab)_2_ (Medac, Hamburg). The binding properties of tribody [(HER2)_2_xCD16] and tribody [(HER2)_2_xCD89] as a control were analyzed by using HER2-positive tumor cells, CD16-expressing immune cells as well as antigen-negative cells as a control. Briefly, 3–5 × 10^5^ cells were incubated with or without purified bispecific antibody derivatives in wash buffer (1% BSA, 0.1% NaN_3_ in PBS) for 30 min at 4°C. Cells were washed twice with washing buffer. After incubation with 10 µg/mL anti-Penta-His™ Alexa Fluor^®^ 488 labeled IgG at 4°C for 30 min, cells were washed and analyzed by a flow cytometer.

For intracellular staining of granzyme B, 5 × 10^5^ cells were washed with staining buffer, fixed and permeabilized with the Cytofix/Cytoperm kit (BD Biosciences). Thereafter, cells were washed twice with Perm/Wash by centrifugation and stained with PE-conjugated anti-granzyme B mAb clone GB11 (BD Biosciences) for 30 min, washed and measured.

All samples were analyzed on a FACS-Calibur or LSR-Fortessa flow cytometer (both from BD Biosciences) using CellQuestPro, Diva, or FlowJo software.

### ^51^Cr-Release Assay

Cytotoxicity against tumor cells was analyzed in a standard 4 h ^51^Cr-release assay with titrated numbers of γδ T effector cells supplemented with 12.5 U/mL IL-2 as described elsewhere (38). To investigate possible increase of cytotoxic activity, cells were treated with 300 nM BrHPP, the indicated concentrations of trastuzumab, tribody [(HER2)_2_xCD16], or tribody [(CD20)_2_xCD16] as a control construct. Supernatants were measured in a MicroBeta Trilux β-counter (Perkin Elmer). Specific lysis was calculated as [(cpm_test_ − cpm_spontaneous_)/(cpm_max_ − cpm_spontaneous_)] × 100, where spontaneous release was determined in medium only and maximal release in Triton-X-100-lysed target cells. Spontaneous release did not exceed 15% of the maximal release.

### Real-Time Cell Analyzer (RTCA)

As an alternative assay to measure cytotoxicity against adherent cells a real-time cell analyzer (RTCA, xCELLigence, ACEA, San Diego, CA, USA) was used as described elsewhere (35, 43, 46). Briefly, 5,000–15,000 adherent tumor cells/well were added to 96-well micro-E-plate in complete medium to monitor the impedance of the cells *via* electronic sensors every 5 min for up to 24 h. The impedance of the cells is expressed as an arbitrary unit called cell index (CI) which reflects changes in cellular parameters such as morphological changes (e.g., spreading, adherence), cell proliferation, and cell death. Since the initial adherence in different wells can differ slightly, the CI was normalized to 1 after having reached the linear growth phase and before the addition of constructs, substances, or suspended cells. Then, trastuzumab or tribody [(HER2)_2_xCD16] and corresponding control constructs were added in the previously titrated optimal concentrations. Thereafter, PBL, freshly isolated γδ T cells or NK cells, or short-term activated γδ T cells together with the indicated 12.5–50 U/mL IL-2 were added to the RTCA-single plate (SP) assay (xCELLigence). Where indicated, short-term activated Vδ2 γδ T cells were stimulated with 300 nM BrHPP. When effector cells induced lysis of the tumor cells, the loss of impedance of tumor cells shown as decrease of the normalized CI was analyzed. As positive control for killing, tumor cells were treated with a final concentration of 1% Triton X-100. For the precise analysis of cytotoxicity, the cells were monitored every minute for the indicated time points.

### CD107a-Degranulation Assay and ELISA

Twenty thousand PDAC cells in 96-well microtiter plates (Nunc, Wiesbaden) were cultured overnight. Medium or 1 µg/mL [(HER2)_2_xCD16] or control constructs with or without 300 nM BrHPP together with short-term activated γδ T cells (E/T ratio: 12.5:1) supplemented with 12.5 U/mL rIL-2 were added after 24 h. For CD107a-assay, 10 µL FITC-labeled anti-human CD107a mAb clone H4A3 (50 µg/mL, Biolegend) was added directly, whereas 3 µM monensin was added 1 h after co-culturing the cells. After additional 3 h, γδ T cells were stained with PE-labeled anti-TCRγδ mAb, and analyzed by flow cytometry.

For assessment of granzyme B with ELISA, cell culture supernatants of duplicates were collected after 4 h and stored at −20°C until use. Human granzyme B was measured by a sensitive sandwich ELISA following the procedures outlined by the manufacturer (R&D System, Wiesbaden, Germany).

### Statistical Analysis

The statistical analysis was assessed by Wilcoxon rank sum test or ANOVA using Graph Pad Prism (Graph Pad Software, Inc., La Jolla, CA, USA). Data from at least three independent biological replicates were used to test for normal distribution with the Shapiro–Wilk test (Graph pad Prism) followed by a parametrically *t* test using Microsoft Excel. All statistical tests were two-sided and the level of significance was set at 5%.

## Results

### Differential Trastuzumab-Mediated Lysis of Cancer Cells

**Figure 1:**
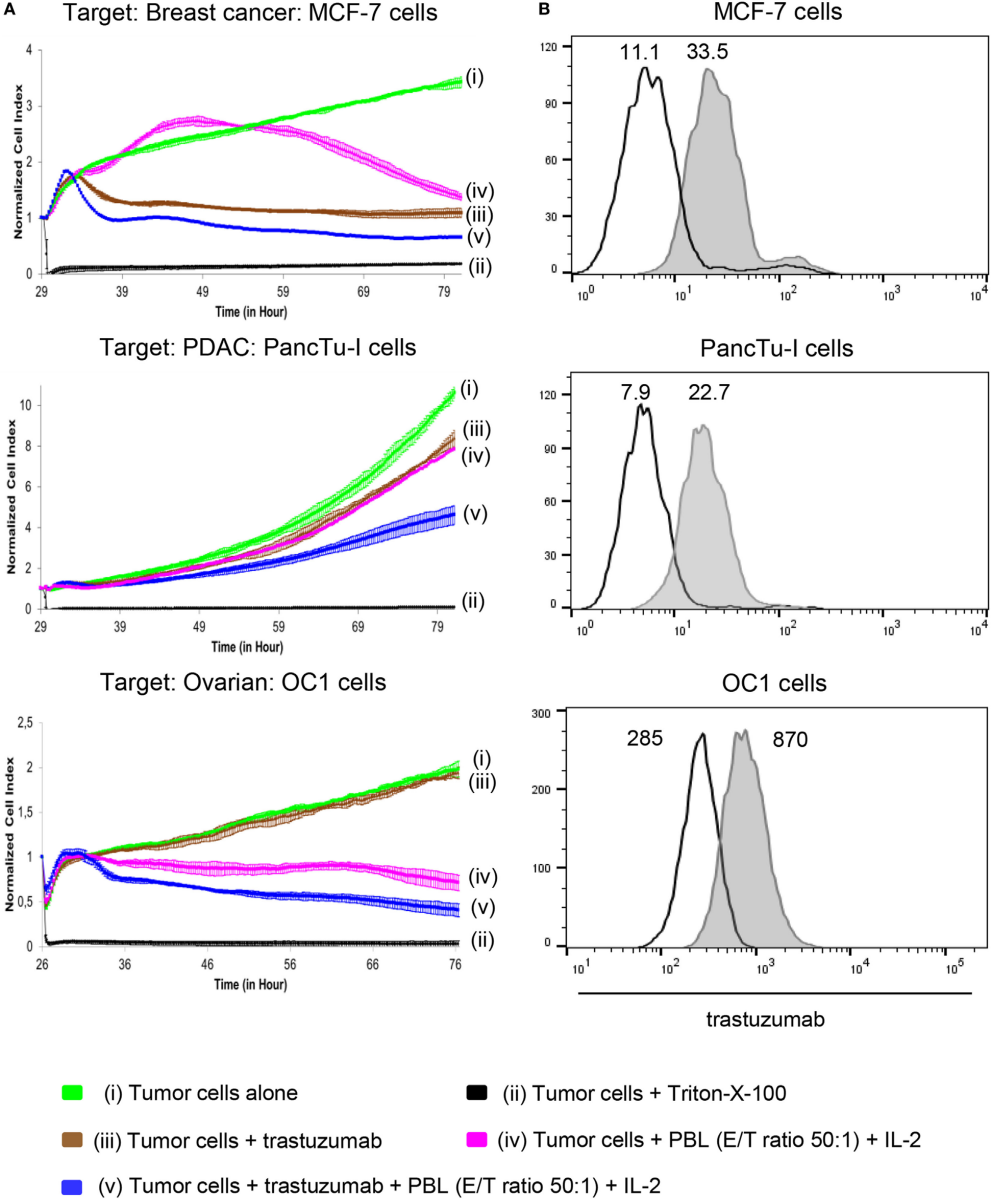
Effect of trastuzumab on breast cancer, pancreatic ductal adenocarcinoma (PDAC), and ovarian cancer (OC) cells with different human epidermal growth factor receptor 2 (HER2) expression. **(A)** 10 × 10^3^ MCF-7 cells, 5 × 10^3^ PancTu-I cells, and 5 × 10^3^ primary OC cells, OC1 [(i), green lines] were cultured in complete medium for 26–29 h on an E-plate covered at the bottom with electronic sensors that measured the impedance of the adherent tumor cells expressed as an arbitrary unit called cell index (CI) every 5 min. The CI was normalized to 1 shortly before the addition of substances as follows: (ii) Triton-X-100 to induce maximal lysis (black line), (iii) 10 µg/mL trastuzumab (brown line), (iv) peripheral blood lymphocytes (PBL) of allogeneic breast cancer, PDAC, or OC patients (E/T ratio 50:1), respectively, together with 50 IU/mL rIL-2 (purple line), or (v) a combination of trastuzumab with PBL (blue line). CI was then measured every minute for additional 40 h. The loss of tumor cell impedance and thus a decrease of CI correlated with lysis of tumor cells. The average of triplicates and SD were calculated; one representative experiment out of four is shown. **(B)** HER2 expression of MCF-7 cells, PancTu-I cells, and primary OC cells OC1 cells was analyzed by staining the cells with 10 µg/mL trastuzumab (gray histograms) and appropriate isotype controls (open black lines) as indicated, followed by appropriate second step Ab and measuring by flow cytometry. Numbers indicate the median fluorescence intensity of the isotype control and staining with trastuzumab, respectively.

In this study, we asked whether the treatment of HER2-expressing PDAC cells or OC cells with trastuzumab in the absence or presence of patients’ PBL can induce tumor cell lysis comparable to that of breast cancer cells. In order to analyze trastuzumab-mediated lysis of HER2-expressing cancer cells by inhibition of HER2 signaling after its homodimerization and ADCC-mediated lysis by FcγRIII (CD16)-expressing cells, such as NK cells or γδ T cells, we applied a RTCA-assay. The RTCA system allows monitoring of cytotoxic activity of a low number of innate and innate-like cells such as NK cells and γδ T cells within PBL, respectively, in real time without the incorporation of labels over time periods of several days. As shown in Figure 1A, the addition of trastuzumab alone induced a strong, but incomplete cell death of breast cancer cell line MCF-7 (Figure 1A), whereas there was almost no lysis of PDAC cell line PancTu-I and primary OC cells OC1 by trastuzumab. The co-culture of tumor cells with IL-2 stimulated allogeneic breast cancer, PDAC, or OC patients’ PBL, respectively, in the absence of trastuzumab induced a delayed lysis of MCF-7 cells, a low lysis rate of PancTu-I cells and an obvious lysis of OC1 cells (Figure 1A) in comparison to the trastuzumab-mediated lysis suggesting an IL-2-mediated activation of PBL effector cells. PBL without IL-2 did not lyse these tumor cells (data not shown). The combination of trastuzumab and PBL enhanced lysis of all applied tumor cells (Figure 1A) compared to trastuzumab alone. This effect was more pronounced in MCF-7- and OC1-cells than in PancTu-I cells and other PDAC cells such as Panc1 co-cultured with allogeneic PBL from HDs (Figure S1A in Supplementary Material, upper figure). In contrast, the OC cell line SK-OV-3 or primary OC cells OC11, which highly expressed HER2 (Figure S1B in Supplementary Material), but not IGROV-1 cells (data not shown), were lysed very efficiently after co-culture with allogeneic PBL of HDs in the presence of IL-2 and trastuzumab suggesting a higher heterogeneity within OC cells (Figure 1A; Figure S1A in Supplementary Material). The differential intensity of lysis induced by trastuzumab alone was not due to a heterogenous HER2 expression as shown by similar staining patterns of the tumor cells with trastzumab (Figure 1B; Figure S1B in Supplementary Material). Taken together, in the presence of patients’ PBL trastuzumab induced lysis of HER2-expressing tumor cells but was unable to eradicate all target cells. In further experiments, we asked for possible reasons for this and strategies to further enhance cytotoxicity by cells of the innate and innate-like immunity.

### Characterization of Immune Cells of Tumor Patients Which Can Be Engaged by Trastuzumab

**Figure 2:**
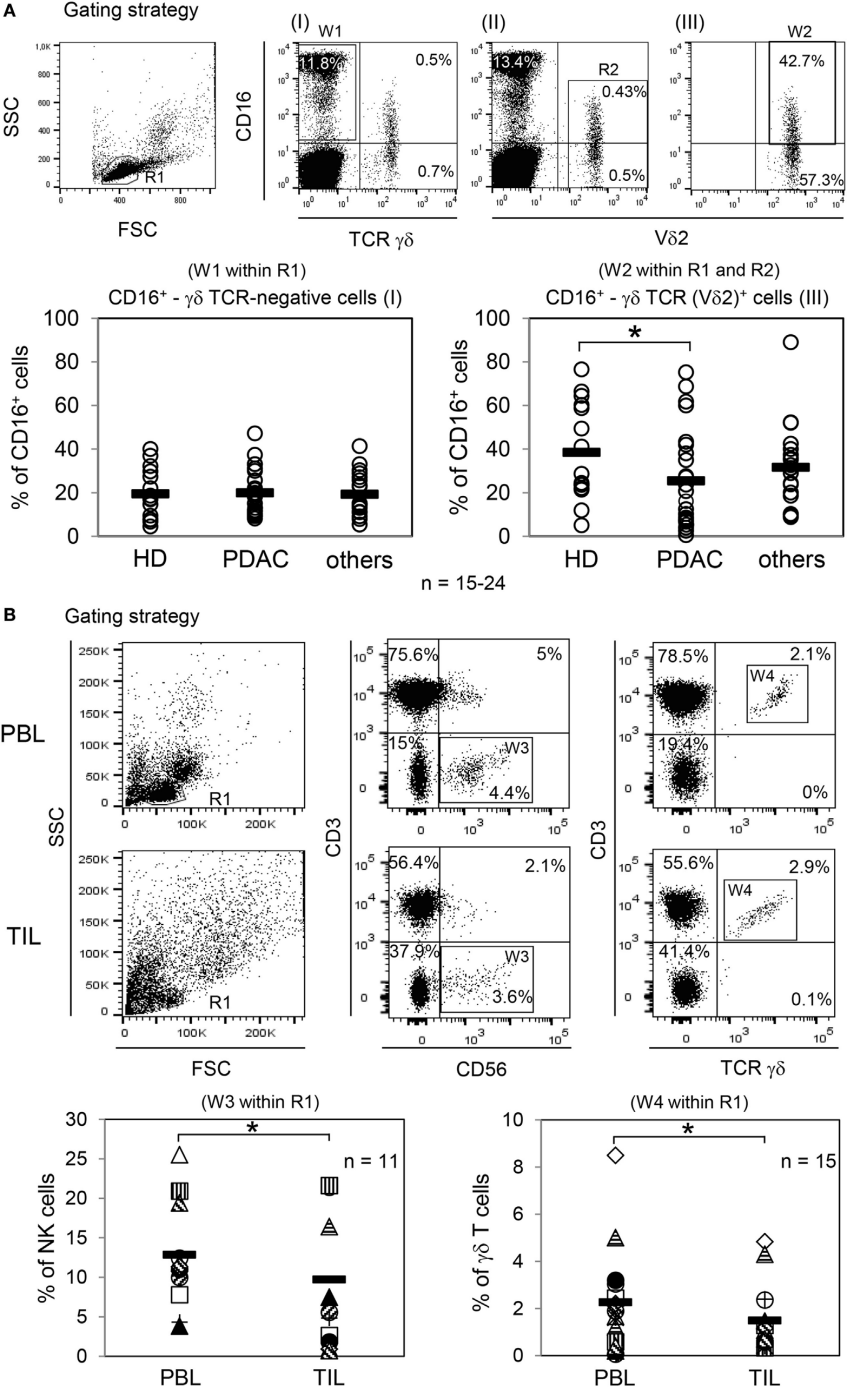
Analysis of CD16 expression within peripheral blood lymphocytes (PBL) and distribution of natural killer (NK) cells and γδ T cells within PBL and tumor-infiltrating lymphocytes (TIL) of ovarian cancer (OC) patients. **(A)** Gating strategy with one healthy donor (HD) for determination of CD16^+^ γδ T cell-negative or -positive cells. CD16^+^ γδ TCR-negative cells were calculated within lymphocytes (R1; FSC/SSC) by pan T cell receptor (TCR) γδ-negative and CD16-positive expression (W1; I). CD16^+^ TCR γδ (Vδ2)-positive T cells were determined within lymphocytes (R1; FSC/SSC) by CD16-positive as well as by pan TCR γδ-positive (I) and Vδ2-positive (II) cells. For precise CD16 expression on Vδ2-positive T cells, an additional gate (R2; II) was set and the expression was calculated in the logical gate of “R1 AND R2” (W2; III). In parallel, CD3 expression of all analyzed γδ T cells was determined (data not shown). The relative percentage of CD16-expressing γδ T cell-negative cells [NK cells, (W1 within R1)] and TCR Vδ2 γδ-positive T cells (W2 within R1 and R2) were analyzed from PBL of age-matched HDs (HD, *n* = 24, 12 females, 12 males; age 65 ± 10 years), from PDAC patients (*n* = 24, 12 females, 12 males; age 64 ± 11 years), and from patients with other cancer diseases (*n* = 15, age 64 ± 11 years). Each symbol represents the data of one donor, and the thick bars represent the mean value of different experiments. Significances were performed by the Wilcoxon rank sum test and are shown as *P* value; *P < 0.05. **(B)** Gating strategy of NK cells and γδ T cells within PBL or TIL of one OC patient. NK cells were defined by CD3-negative and CD56^+^ cells (W3) within R1 (FSC/SSC) and γδ T cells by CD3^+^ pan TCR γδ^+^ cells (W4) within R1. The relative percentage of NK cells (*n* = 11; age 57 ± 10) is shown in “W3 within R1” and for γδ T cells (*n* = 15; age 56 ± 13) in “W4 within R1.” Each symbol presents the data of one donor. Mean values of different experiments are indicated (thick bars). Significances were assessed by the Wilcoxon rank sum test and are shown as *P* value, *P < 0.05.

Besides varying direct effects of trastuzumab on the different tumor cells, one possible additional explanation for the weak lysis of PDAC cell lines by PBL from cancer patients is our observation of a significantly decreased CD16 expression on Vδ2-positive γδ T cells within the PBL of PDAC patients (Figure 2A, right panel), in contrast to TCR γδ-negative NK cells (Figure 2A, left panel), which was not present in age-matched HDs or patients with other different (cancer) diseases (shortly before surgery, listed in Section “Materials and Methods”) (Figure 2A). Of note expression of other activating Fcγ receptors, i.e., Fcγ RI (CD64) and Fcγ RIIA (CD32A) on γδ T cells were not observed (data not shown). Independent of our *in vitro* assay studies with PBL and tumor cells, a low number of CD16-expressing immune cells within the tumors can be responsible for a reduced response toward trastuzumab in tumor patients. Thus, we exemplarily analyzed fresh tumor material of advanced OC patients, where the majority of the analyzed tumors expressed HER2 and compared the distribution of NK cells and γδ T cells within the tumors and the blood of these patients (Figure 2B). Interestingly, a significantly reduced number of NK- and γδ-T cells within TIL compared to PBL was observed (Figure 2B).

### Bispecific Antibody as an Alternative to Trastuzumab

**Figure 3:**
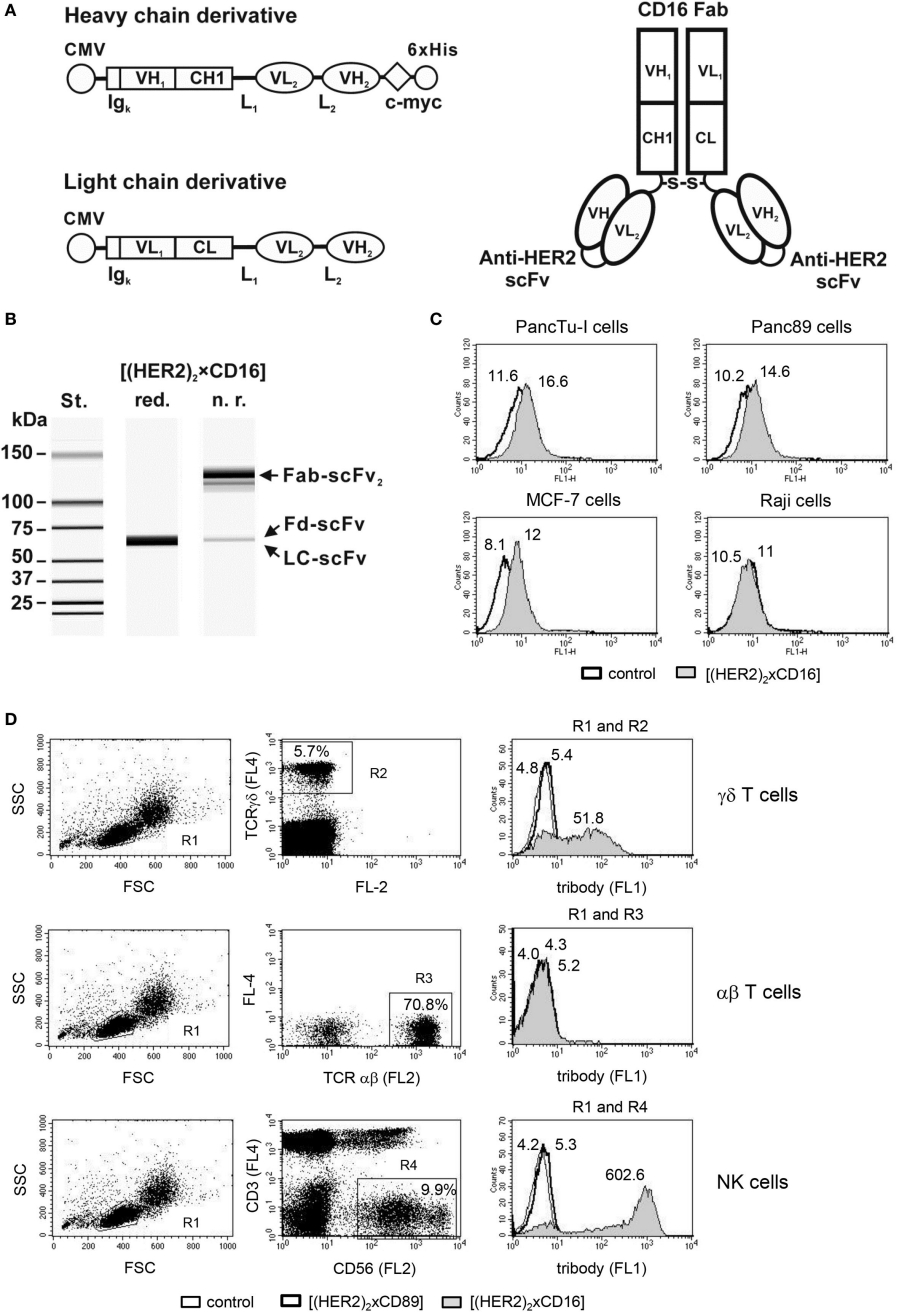
Design and purification of [(HER2)_2_xCD16]. **(A)** Scheme of the expression cassettes for (left) the tribody protein and (right) the assembled tribody molecule. CMV, cytomegalovirus immediate early promoter; Ig_κ_, murine Ig kappa secretion leader; VH_1_, VL_1_, and CH1, CL, sequences coding for the human immunglobulin (Ig) variable (V) or constant (C) heavy and light chain regions from the CD16 mAb, respectively; VH_2_, VL_2_, sequences coding for the variable heavy and light chain regions building a scFv with specificity for HER2; L_1_, L_2_, sequence coding for a 15 amino acid flexible linker (G_4_S)_3_ and a 20 amino acid flexible linker (G_4_S)_4_, respectively; c-myc and 6xHis, sequence coding for the c-myc epitope and a hexahistidine tag, respectively. S-S, disulfide bond. **(B)** Evaluation of the purity of the [(HER2)_2_xCD16] tribody by capillary electrophoresis; Fd, fragment difficult; LC, light chain. Specific binding of **(C)** tumor cells (e.g., PancTu-I, Panc89, MCF-7, and Raji) and **(D)** γδ T and NK cells within PBL and lack of binding to αβ T cells within PBL was analyzed by flow cytometry. Representative experiments out of three analyzed are presented. [**(D)**, left and middle panel] The percentage of γδ T cells were defined by pan TCR γδ^+^ cells (R2), αβ T cells by pan TCR αβ^+^ cells (R3), and NK cells by CD3-negative and CD56^+^ cells (R4), respectively, all within the lymphocyte gate R1 (FSC/SSC). [**(D)**, right panel] The median fluorescence intensity of isotype or tribody staining was presented by histogram blots for γδ T cells (R1 and R2), αβ T cells (R1 and R3), or for NK cells (R1and R4). Numbers indicate the median fluorescence intensity of the isotype control [bold line in **(C)** and thin line in **(D)** as indicated] and staining with [(HER2)_2_xCD89] [bold line in **(D)**] or [(HER2)_2_xCD16] [gray filled in **(C,D)**] respectively.

As trastuzumab did not optimally inhibit tumor cell proliferation, we employed a bispecific antibody in the tribody format (Figure 3A). [(HER2)_2_xCD16] consists of a CD16 Fab fragment and two single chain fragments variable (scFv), which were derived from trastuzumab variable regions and which were genetically fused with a flexible linker to the CH1- or CL-domains of the CD16 Fab. [(HER2)_2_xCD16] was transiently expressed in Lenti-X 293 T cells, purified, and analyzed by capillary electrophoresis (Figure 3B). Tribody [(HER2)_2_xCD16] specifically bound HER2-positive tumor cells (Figure 3C) and CD16-expressing NK cells as well as γδ T cells (Figure 3D), but not antigen-negative cells such as Raji or αβ T cells (Figures 3C,D). In addition, tribody [(HER2)_2_xCD89] was used as a control, which did not bind to CD89-negative NK cells, γδ-, or αβ-T cells (Figure 3D). The cytotoxic activity triggered by the tribody [(HER2)_2_xCD16] was initially analyzed at varying concentrations and varying effector to target cell ratios (E/T ratio) (data not shown and following Figures). We measured a saturating concentration of tribody [(HER2)_2_xCD16] between 0.1 and 1 µg/mL. In contrast, the saturating concentration of trastuzumab was 10 µg/mL.

### Enhanced PBL-Mediated Lysis of HER2-Expressing Tumor Cells by Tribody [(HER2)_2_xCD16] in Comparison to Trastuzumab

**Figure 4:**
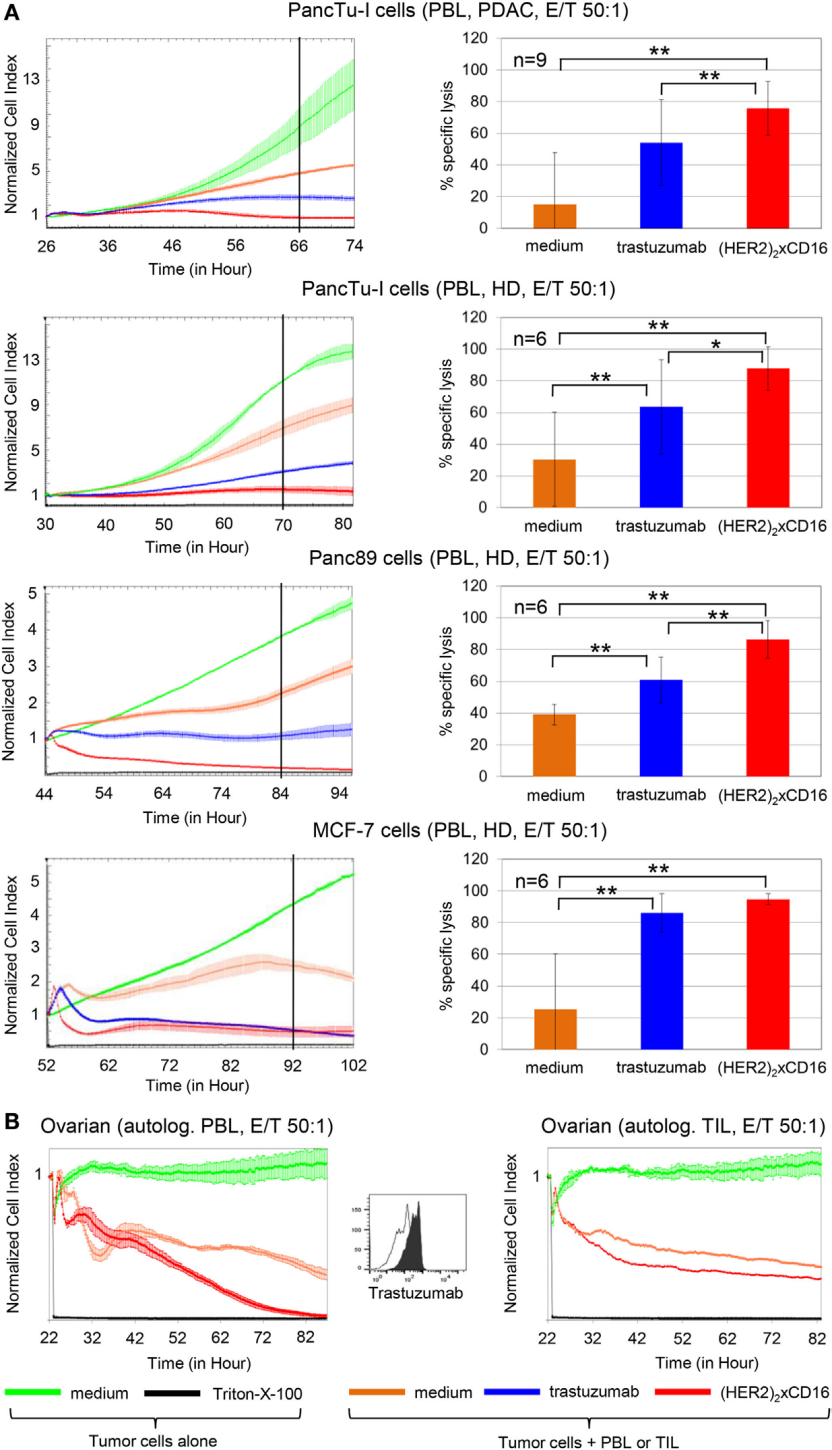
Tribody [(HER2)_2_xCD16] increased cytotoxicity compared to trastuzumab. **(A)** After culturing 5 × 10^3^ pancreatic ductal adenocarcinoma (PDAC) cells (PancTu-I- and Panc89 cells), 10 × 10^3^ breast cancer cells (MCF-7 cells) overnight, cells were cultured without or with allogeneic peripheral blood lymphocytes (PBL) at an E/T ratio of 50:1 in the presence of 50 IU/mL rIL-2 with medium (orange line), 10 µg/mL trastuzumab (blue line), or 1 µg/mL tribody [(HER2)_2_xCD16] (red line). As control cells were left untreated (green line) or treated with Triton-X-100 (black line). **(B)** 5 × 10^3^ primary ovarian cancer (OC) cells OC11 had been cultured overnight in medium before the addition of medium control (green line) or Triton-X-100 (black line) in the absence of effector cells. Autologous PBL or TIL of human epidermal growth factor receptor 2 (HER2)-expressing OC patient OC11 cultivated with 50 IU/mL rIL-2 were added at an E/T ratio of 50:1 alone (orange line) or with 1 µg/mL tribody [(HER2)_2_xCD16] (red line). **(A,B)** The cell index was analyzed in 5 min steps over ~24–52 h and after normalization to 1 (after addition of PBL ± substances) in 1 min steps for >50 h as indicated. The average of three replicates with SD is presented for each tumor cell line with PBL **(A,B)** or TIL [**(B)**, right figure for OC] of one representative healthy donor or cancer patients (PDAC, OC, left panel) in independent experiments. For validation, experiments were replicated several times under equal conditions using different PBL of different donors in independent experiments (right panel). The cytotoxic capacity of PBL against the indicated tumor cells in the presence of medium (orange bars), 10 µg/mL trastuzumab (blue bars), or 1 µg/mL tribody [(HER2)_2_xCD16] (red bars) was calculated 40 h (black line, left panel) after addition of effector cells (PBL) as % of specific lysis compared to control sample (green line) and maximal lysis (black line). Statistical analysis was performed by *t* test. Significances are shown as *P* value; *P < 0.05 and **P < 0.01.

To compare the cytotoxic potential of [(HER2)_2_xCD16] tribody with that of trastuzumab, we initially analyzed freshly isolated PBL from different PDAC patients (*n* = 9) and HDs (*n* = 6) that had been co-cultured with HER2-expressing PDAC cells (PancTu-I- or Panc89 cells) or breast cancer cells (MCF-7 cells) in the RTCA system (Figure 4A). Previously titrated saturating concentrations of 10 µg/mL trastuzumab and 1 µg/mL of tribody [(HER2)_2_xCD16] together with IL-2 were applied (Figure 4A). Although the concentration of tribody [(HER2)_2_xCD16] was 10-fold lower than that of trastuzumab, the tribody significantly and more potently enhanced the PBL-mediated lysis of HER2-expressing tumor cells (Figure 4A).

Moreover, PBL or TIL of patient OC11 were co-cultured with autologous primary ovarian tumor cells OC11 in the presence or absence of 1 µg/mL tribody [(HER2)_2_xCD16] together with IL-2 (Figure 4B). Interestingly, lysis of HER2-expressing primary ovarian tumor cells by autologous PBL or TIL was also significantly increased after the addition of tribody [(HER2)_2_xCD16] (Figure 4B). The TIL-mediated lysis of autologous primary ovarian tumor cells was not complete, but very impressive due to the very low percentage of <0.5% NK cells and 0.9% γδ T cells compared to 5% NK cells and 1.5% γδ T cells within the PBL (Figure 4B; Figure 2B filled circle). Further, tribody [(HER2)_2_xCD16] alone or control constructs such as tribody [(HER2)_2_xCD89] which has no specificity for NK cells or γδ T cells or tribody [(CD20)_2_xCD16] targeting CD20 being not expressed on the applied tumor cells, did not trigger target cell lysis (Figure S2 in Supplementary Material).

### NK Cells as Well as γδ T Cells Are Activated by Tribody [(HER2)_2_xCD16]

**Figure 5:**
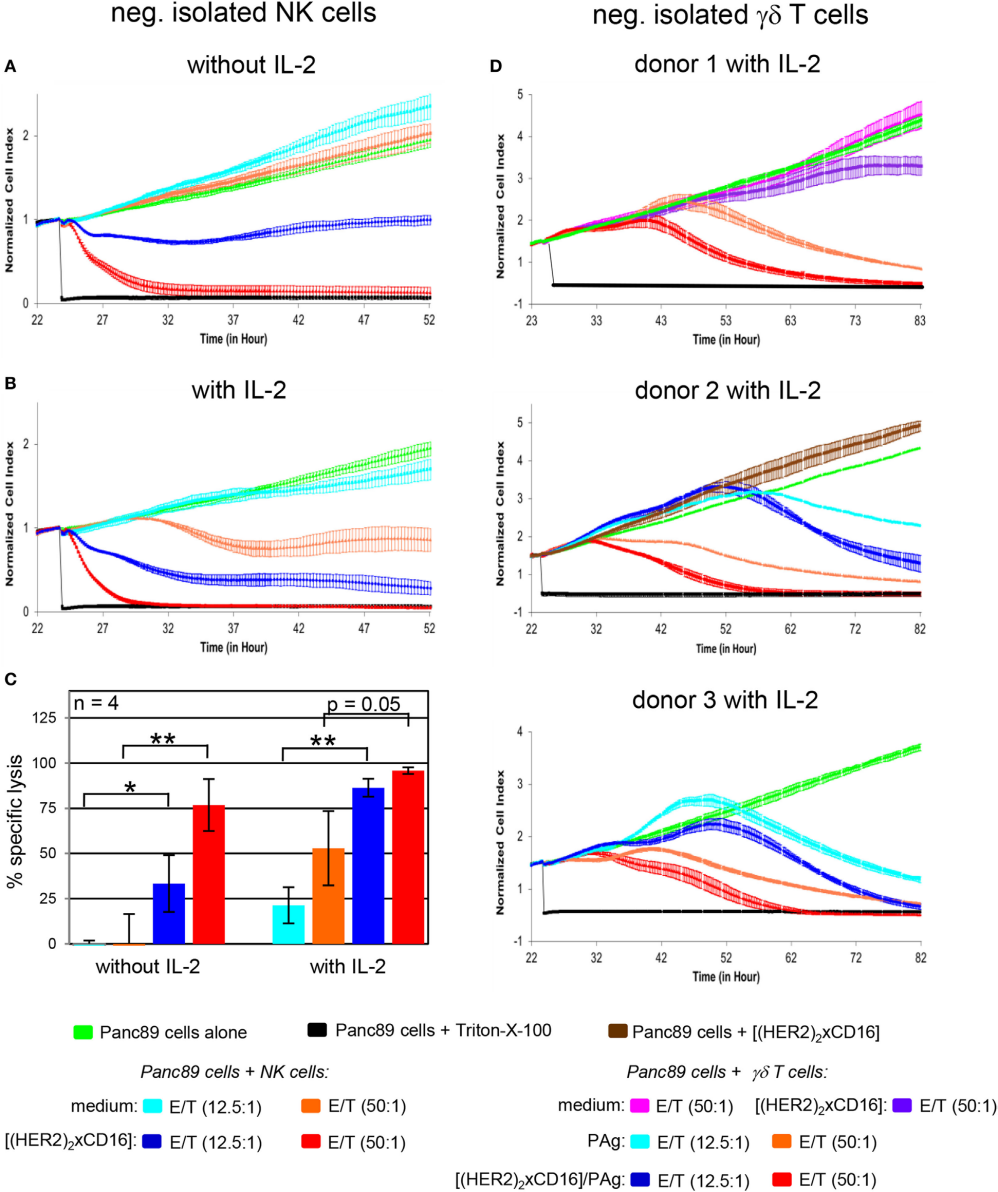
Enhancement of freshly isolated natural killer (NK) cell- and γδ T cell-mediated lysis of Panc89 cells by tribody [(HER2)_2_xCD16]. 5 × 10^3^ Panc89 cells were seeded overnight in complete medium. Cell index was analyzed in 5 min steps over ~22 h. After overnight adherence, Panc89 cells were cultured with additional complete medium (green line), 1 µg/mL tribody [(HER2)_2_xCD16] (brown line), or positive control Triton-X-100 (black line). Additionally, Panc89 cells were co-cultured with **(A–C)** allogeneic negatively, freshly isolated NK cells or **(D)** γδ T cells at the indicated E/T ratio with or without 50 IU/mL rIL-2 (as indicated) in the presence of medium [light blue line (E/T ratio 12.5:1); orange line (E/T ratio 50:1)] or 1 µg/mL tribody [(HER2)_2_xCD16] [dark blue line (E/T ratio 12.5:1); red line (E/T ratio 50:1)] and for γδ T cells with 300 nM phosphorylated antigen bromohydrin-pyrophosphate (BrHPP) or without BrHPP in medium (pink line) or 1 µg/mL tribody [(HER2)_2_xCD16] at an E/T ratio 50:1. Lysis of tumor cells was measured after normalization to 1 in 1 min steps for >30 h as indicated. **(A,B,D)** The average of three replicates with SD is represented for each tumor cell line with effector cells of one representative healthy donor (HD) in independent experiments. **(C)** Several replications of the experiments under equal conditions using different NK cells of different donors in independent experiments were performed. The NK cell cytotoxicity against Panc89 cells was calculated 30 h after addition of NK cells without or with IL-2. The % of specific lysis was calculated by comparing measured samples to control sample (green line) and maximal lysis (black line). Statistical analysis was performed by *t* test. Significances are shown as *P* value; *P < 0.05 and **P < 0.01.

To analyze whether the cytotoxicity of both NK cells and γδ T cells can be enhanced by the tribody [(HER2)_2_xCD16], we magnetically isolated both cell populations from PBL of HDs. HER2-expressing PDAC cells such as Panc89 cells were used as targets to analyze the effect of the tribody [(HER2)_2_xCD16] on freshly isolated NK cell- or γδ T cell cytotoxicity in several RTCA-assays. We observed that the tribody [(HER2)_2_xCD16] enhanced cytotoxic activity of freshly, negatively isolated NK cells which was dependent on the number of NK cells as expected (Figures 5A–C). At a higher E/T ratio all tumor cells were lysed. At lower E/T ratios tribody [(HER2)_2_xCD16] triggered lysis was more pronounced in the presence of IL-2 (Figures 5B,C). Regarding a reduced percentage of innate immune cells at the tumor site as described for NK cells (Figure 2B), a parallel activation of additional innate-like immune cells can be beneficial. For instance, freshly, negatively isolated γδ T cells can be activated *via* the TCR through their selective antigens [e.g., phosphorylated antigens (PAg)] in the presence of exogenous IL-2 to kill HER2-expressing Panc89 cells (Figure 5D, light blue and orange line indicating different E/T ratios), which can be further increased by the addition of tribody [(HER2)_2_xCD16] (Figure 5D, dark blue and red line indicating different E/T ratios). In contrast to NK cells, the addition of exogenous IL-2 as well as the TCR engagement is absolutely essential for inducing optimal cytotoxic activity of freshly isolated γδ T cells (Figure 5D, upper panel, pink and purple line indicating different E/T ratios). Tribody [(HER2)_2_xCD16] alone (central Figure 5D, brown line) and control constructs such as tribody [(HER2)_2_xCD89] or tribody [(CD20)_2_xCD16] did not trigger target lysis (Figure S2 in Supplementary Material).

### Tribody [(HER2)_2_xCD16] Increased Cytotoxic Activity of γδ T Cells Suitable for Adoptive Cell Transfer

**Figure 6:**
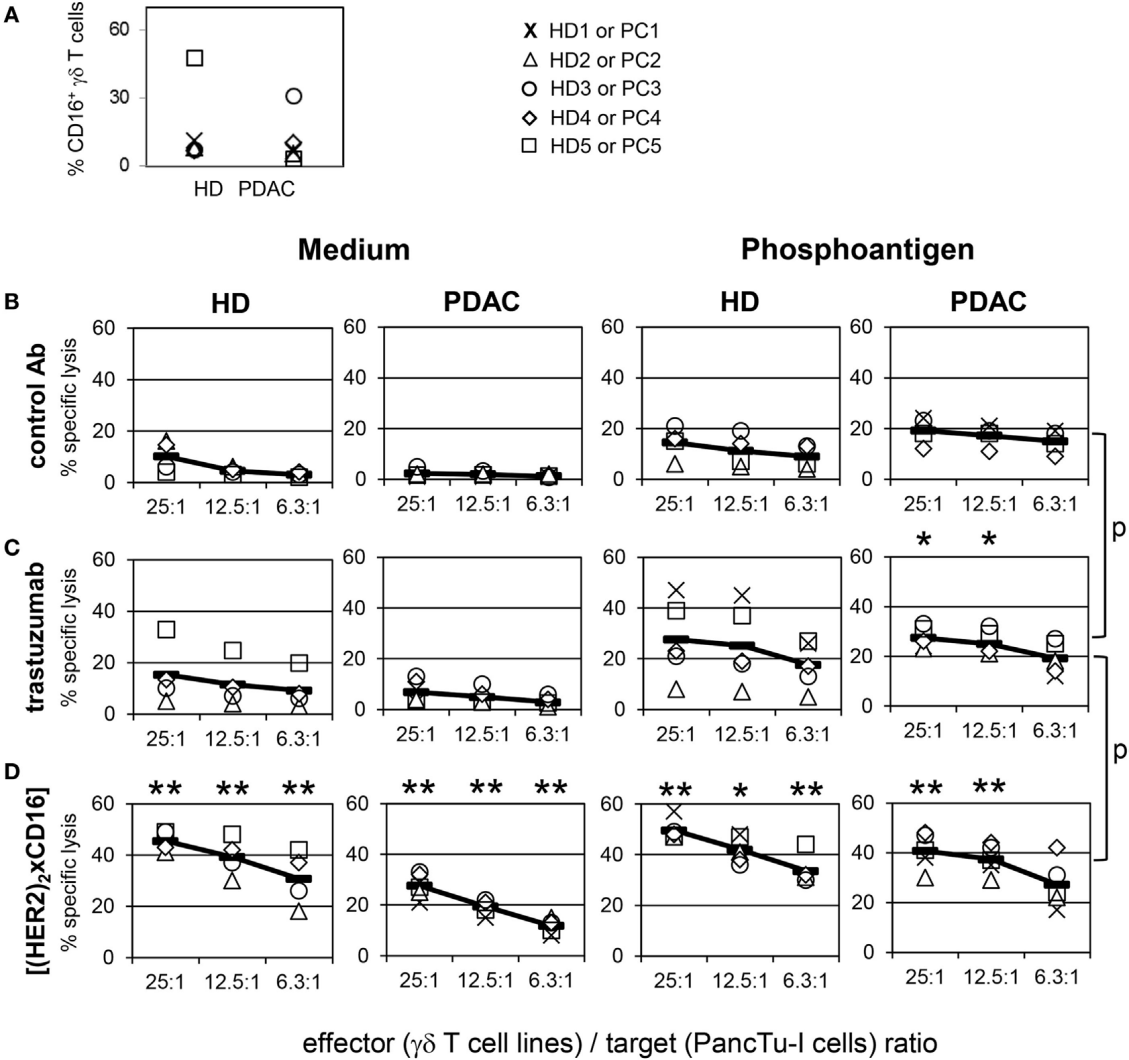
Enhancement of γδ T cell-mediated pancreatic ductal adenocarcinoma (PDAC) lysis by tribody [(HER2)_2_xCD16]. **(A)** Vγ9Vδ2 γδ T cell lines of healthy donors (HDs) or PDAC patients were stained with anti-CD16 monoclonal antibodies (% CD16^+^ γδ T cells) or cultured with **(B)** control Ab, **(C)** 10 µg/mL trastuzumab, or **(D)** 1 µg/mL tribody [(HER2)_2_xCD16] in complete medium (left panel) or with 300 nM phosphoantigen bromohydrin-pyrophosphate (right panel) as indicated. **(B–D)** γδ T cell lines at the indicated E/T ratios were added to ^51^Cr-labeled PancTu-I cells for 4 h. Each symbol represents the mean value of triplicate assays of one donor. Representative results from five different HDs and five different PDAC patients are shown. Statistical analysis was performed by *t* test. Significances are shown as *P* value; *P < 0.05 and **P < 0.01.

In general bsAb can be applied in cancer immunotherapy in various clinical settings. Besides application as single agent, they can be combined in regimens including adoptive cell transfer. Patient-derived γδ T cells expanded with their selective antigens and IL-2 *in vitro* might be combined with bsAb to enhance cytotoxic activity of transferred γδ T cells. The latter approach requires an examination of the cytotoxic activity of short-term expanded γδ T cells in comparison to freshly isolated ones. As shown in ^51^chromium-release experiments, we observed a limited cytotoxic capacity of short-term expanded γδ T cells from HDs (*n* = 5) as well as from PDAC patients (*n* = 5) against the PDAC cell line PancTu-I (Figure 6B, medium, left two panels). The limited cytotoxicity of short-term expanded γδ T cells was somewhat enhanced by the addition of their selective antigens such as PAg (Figure 6B, phosphoantigen, right two panels), whereas the addition of trastuzumab only weakly increased the cytotoxicity of these γδ T cells (Figure 6C, medium). Lysis induced by trastuzumab (Figure 6C, medium) correlated with the CD16 expression on the surface of the γδ T cells (Figure 6A). Thus, short-term expanded γδ T cells of HD donor 5 (Figure 6A, quadrat) and PDAC donor 3 (Figure 6A, circle), which both had a high percentage of CD16-positive cells, exerted an enhanced cytotoxic activity (Figure 6C, medium). In contrast, short-term expanded γδ T cells of other donors, which expressed lower numbers of CD16 (Figure 6A), were only slightly affected (Figure 6C, medium). Similar to the γδ T cells within the PBL, we screened the CD16 expression on all established short-term expanded γδ T cells of HD (*n* = 43) as well as of PDAC patients (*n* = 20) which were used in different experiments in this studies (Figure S3 in Supplementary Material). In general, we observed a high variability in CD16 expression between 1 and 70% which drastically influenced the effectiveness of trastuzumab (Figure S3 in Supplementary Material; Figure 6C). Although we observed that the combination of trastuzumab and PAg BrHPP enhanced γδ T cell-mediated killing of PancTu-I cells (Figure 6C, phosphoantigen), the application of tribody [(HER2)_2_xCD16] was more potent in enhancing γδ T cell-mediated killing (Figure 6D, medium) than trastuzumab (Figure 6C, medium) presumably due to the higher CD16 binding affinity. Interestingly, the combination of tribody [(HER2)_2_xCD16] and BrHPP enhanced the cytotoxic activity of γδ T cells from PDAC patients more prominent than γδ T cells from HDs (Figure 6D, phosphoantigen). This can be explained by the lower CD16 expression on γδ T cell lines of PDAC patients (Figure 6A). Importantly, it demonstrates the effectiveness of tribody [(HER2)_2_xCD16] on low CD16-expressing cells.

In another assay with additional short-term expanded γδ T cell lines of three different donors as effector cells and HER2-expressing PancTu-I cells, we analyzed in parallel HER2-negative, CD20-positive Raji tumor cell line as target cells to prove specificity of the tribody [(HER2)_2_xCD16]. Tribody [(HER2)_2_xCD16] triggered lysis of HER2-expressing tumor cells, but not of HER2-negative Raji cells, which, however, were efficiently killed in the presence of PAg BrHPP or tribody [(CD20)_2_xCD16] (Figure S4 in Supplementary Material).

**Figure 7:**
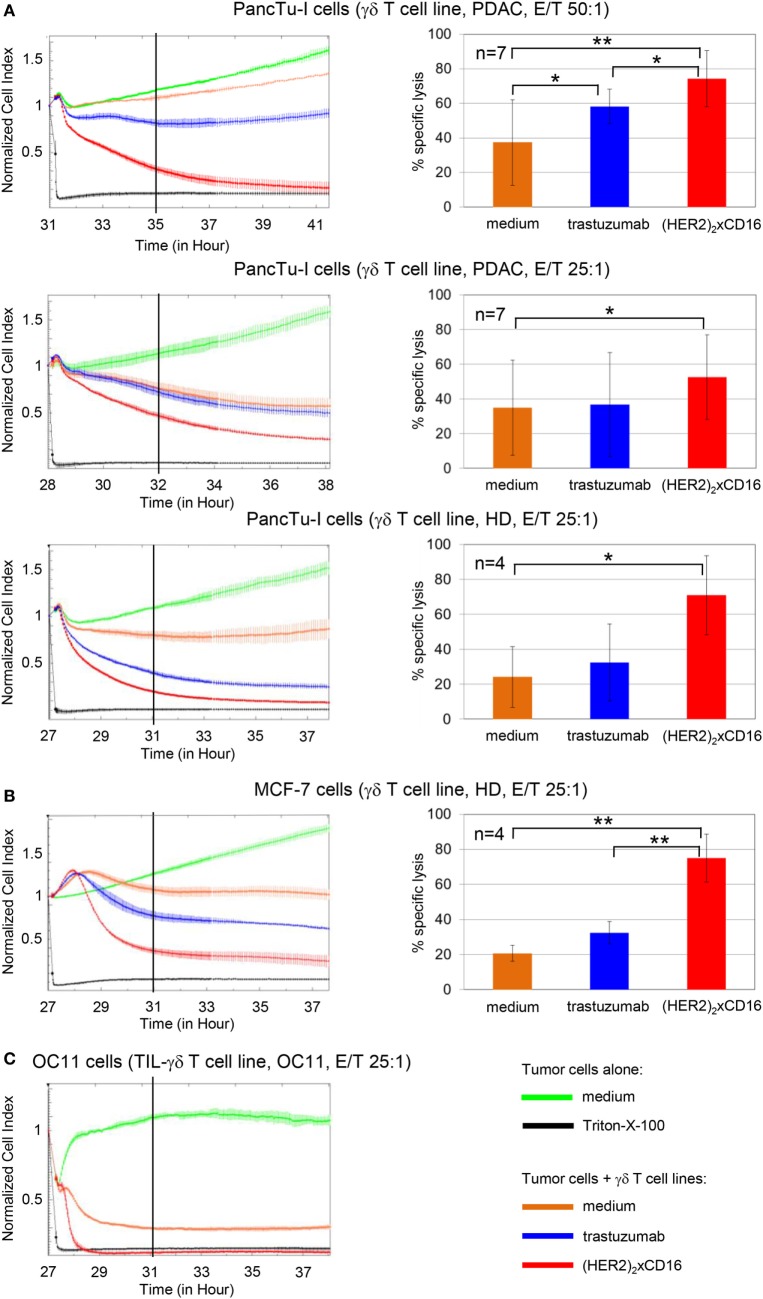
Enhanced cytotoxicity of tribody [(HER2)_2_xCD16] compared to trastuzumab. 5 × 10^3^ PDAC cells (PancTu-I and Panc89 cells), 10 × 10^3^ breast cancer cells (MCF-7 cells), or 5 × 10^3^ ovarain cancer (OC) OC11 cells were seeded overnight. Cell index was analyzed in 5 min steps over ~27–31 h. After reaching the linear growth phase, tumor cells were co-cultured with medium (green line) or with **(A,B)** allogeneic or **(C)** autologous Vγ9Vδ2 T cell lines at the indicated E/T ratio with 12.5 IU/mL rIL-2 in the presence of medium (orange line), 10 µg/mL trastuzumab (blue line), or 1 µg/mL [(HER2)_2_xCD16] tribody (red line). Lysis of tumor cells was measured after normalization to 1 in 1 min steps for >10 h as indicated and compared to maximal lysis (black line). The average of three replicates with SD is represented for each tumor cell line with allogeneic Vγ9Vδ2 T cell lines of one representative healthy donor or cancer patients (PDAC) or autologous Vγ9Vδ2 T cell line established out of the TIL of patient OC11 (left panel) in independent experiments. Several replications of the experiments using different Vγ9Vδ2 T cell lines of different donors in independent experiments were performed (right panel). The cytotoxicity of Vγ9Vδ2 T cell lines against the indicated tumor cells in the presence of medium (orange bars), 10 µg/mL trastuzumab (blue bars), or 1 µg/mL tribody [(HER2)_2_xCD16] (red bars) was calculated 4 h after addition of Vγ9Vδ2 T cell lines. The % of specific lysis was calculated by comparing measured samples to control sample (green line) and maximal lysis (black line). Statistical analysis was performed by *t* test. Significances are shown as *P* value; *P < 0.05 and **P < 0.01.

In addition, the results of the ^51^Cr-release assay for PancTu-I cells were confirmed by the RTCA-assay (Figure 7). Again, the tribody [(HER2)_2_xCD16] enhanced the γδ T cell-mediated cytotoxicity against PancTu-I cells at different E/T ratios more extensively than trastuzumab independent of whether short-term expanded γδ T cells from PDAC patients (*n* = 7) or from HDs (*n* = 4) were used (Figure 7A). Similar to the results with PancTu-I cells, we observed that γδ T cell cytotoxicity against other HER2-expressing tumor cells such as established breast cancer cell line MCF-7 (Figure 7B) or autologous primary OC cells OC11 (Figure 7C) was increased. The lysis of the latter one was enhanced after addition of autologous short-term expanded γδ T cells isolated from TIL together with tribody [(HER2)_2_xCD16]. As expected, the control constructs had no cytolytic effect (data not shown). We observed no significant enhancement of γδ T cell cytotoxicity by the tribody [(HER2)_2_xCD16] compared to trastuzumab when we used a high E/T ratio and Panc89 cells as target cells, which were lysed more efficiently than other PDAC cells (e.g., PancTu-I- or Panc1 cells; Figure S5 in Supplementary Material). Interestingly, a reduced lysis of Panc89 cells by short-term expanded γδ T cells, which was due to a lower E/T ratio (5:1) was more effective with tribody [(HER2)_2_xCD16] than with trastuzumab, although γδ T cells from some donors expressed CD16 very weakly at the cell surface (Figures S3 and S5 in Supplementary Material). Enhanced γδ T cell-mediated lysis with tribody [(HER2)_2_xCD16] was also observed at a lower E/T ratio when other HER2-expressing tumor cells such as the esophageal cancer cell line OE33 were used (Figure S5 in Supplementary Material).

### Enhanced γδ T Cell Cytotoxicity Against HER2-Expressing Tumor Cells by Tribody [(HER2)_2_xCD16] Is Mediated by Cytolytic Granules

**Figure 8:**
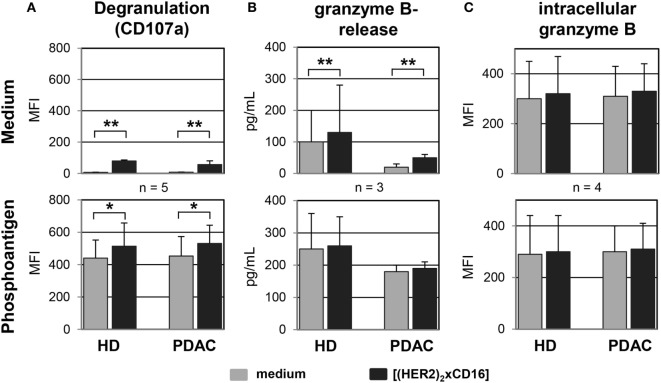
Tribody [(HER2)_2_xCD16] induces an enhanced degranulation and release of granzyme B. Short-term expanded γδ T cells were co-cultured with PancTu-I cells (E/T ratio of 12.5:1) in medium (upper panel) or 300 nM phosphoantigen bromohydrin-pyrophosphate (lower panel) in the presence of medium or 1 µg/mL of the tribody [(HER2)_2_xCD16] and **(A)** degranulation was analyzed by staining γδ T cells with anti-Vδ2 TCR and anti-CD107a monoclonal antibodies and then determining the median fluorescence intensity (MFI) of CD107a on Vδ2 TCR-expressing γδ T cells using flow cytometry or by **(B)** measuring the release of granzyme B in the supernatants using specific ELISAs or **(C)** the intracellular MFI of granzyme B using a flow cytometer after 4 h of co-culturing. Result ± SD of three donors is shown. Statistical analysis was performed by *t* test. Significance is presented as *P < 0.05 and **P < 0.01.

Besides the aspect that several PDAC cells are almost resistant to the CD95- or TRAILR-induced cell death, γδ T cells mainly mediate their cytotoxic activity through the release of cytolytic granules (47). As shown in Figure 8, the CD107a-degranulation assay revealed a significantly enhanced exocytosis after application of tribody [(HER2)_2_xCD16] to short-term expanded γδ T cells from HDs or PDAC patients co-cultured with PancTu-I cells which was accompanied by an increase in granzyme B release (Figures 8A,B). Degranulation was *per se* higher after PAg-stimulation, but was further significantly enhanced after additional application tribody [(HER2)_2_xCD16] (Figure 8A). Interestingly, the intracellular content of granzyme B was not influenced by the treatment with tribody [(HER2)_2_xCD16] suggesting a permanent intracellular production of granzyme B (Figure 8C).

## Discussion

In this study, the tribody [(HER2)_2_xCD16] efficiently enhanced the cytotoxic activity of NK cells and γδ T cells of healthy persons as well as of cancer patients against HER2-expressing tumor cells including breast-, pancreatic-, ovarian-, and esophageal cancer cells. While NK cells can be activated directly by tribody [(HER2)_2_xCD16], the addition of exogenous IL-2 as well as the TCR engagement is essential for inducing cytotoxicity in resting γδ T cells pointing to a pre-activation of γδ T cells by their selective antigens (e.g., PAg or n-BP) *in vivo* or an adoptive transfer of short-term activated γδ T cells together with the tribody as potential therapeutic strategies.

In contrast to trastuzumab, tribody [(HER2)_2_xCD16] enhanced the cytotoxic activity even of CD16 low-expressing γδ T cells of PDAC patients together with their selective antigens (e.g., PAg or nBP) and IL-2 against HER2-low-expressing PDAC cell lines. Interestingly, we observed a reduced number of NK cells within TIL of OC patients before surgery and chemotherapy which may explain the superiority of PBL compared to TIL (both from OC patients) against autologous tumor cells. An activation of γδ TIL of OC patient OC11 after neoadjuvant chemotherapy stimulated *via* PAg together with Th1 cytokine IL-2 and tribody [(HER2)_2_xCD16] efficiently increased lysis of autologous HER2-expressing primary OC cells (OC11) suggesting a compensation of the low number of NK cells within TIL by γδ T cell activation. In this context, our own additional data reveal that the absolute γδ T cell number in a cohort of 26 breast cancer patients receiving chemotherapy did not differ from the number before chemotherapy, in contrast to decreased NK cells and αβ T cell numbers (48).

Human epidermal growth factor receptor 2 can be overexpressed in advanced breast, gastric, colorectal, pancreatic, and OC cells (6). In general, HER2-expressing tumors are regarded as biologically aggressive neoplasms frequently associated with chemo-resistance and poor clinical outcome. Therapy with humanized HER2 mAb targeting different members of the epidermal growth factor receptor (EGFR) family combined with cytostatic agents e.g., docetaxel and potentially tyrosine kinase inhibitors such as, e.g., lapatinib or neratanib/afatinib has clearly improved the outcome of patients with metastatic breast or gastric cancer (6, 10–12). Trastuzumab as well as pertuzumab induce cell death *in vitro* by inhibiting the Ras-MAP-kinase- or PI3K/Akt/mTOR pathway in tumors or ADCC (2, 49). In neoadjuvant settings, trastuzumab and pertuzumab in combination with chemotherapy have been shown to improve pathological complete response (pCR) in early or locally advanced breast cancer. In adjuvant settings, trastuzumab with taxane-based chemotherapy is considered standard of care (2, 11, 50–53).

Combining trastuzumab with chemotherapy has been also described in a few clinical trials to significantly improve overall survival compared with chemotherapy alone in HER2-positive advanced gastric and in one clinical trial in metastatic pancreatic cancer (37, 54, 55). In addition, several case reports described an effect of trastuzumab on HER2-expressing uterine serous and high-graded endometrioid tumors varying from complete response to stable disease for 11 months (56–58). However, single drug trastuzumab in HER2-overexpressing OC show only moderate activity (59). Anti-HER2 monotherapy is currently not a standard therapy for OC patients. Several previously unpublished or ongoing phase I or II trial studies (https://clinicaltrials.gov/.) treated OC patients with either trastuzumab combined with chemotherapy (NCT00433407) or HER2 cytotoxic T-cell (CTL) peptide-based vaccine (NCT00194714) or anti-CD3xanti-HER2/neu (HER2Bi) armed anti-CD3 activated T cells (aATC) plus low-dose aldesleukin and sargramostim (NCT02470559). The application of our tribody [(HER2)_2_xCD16] to OC patients could be an option to enhance avidity to HER2-expressing OC cells due to the format with the two HER2-binding sites as well as the possibility to target innate cells (NK cells) as well as cells linking innate and adaptive immunity (γδ T cells) instead of a polyclonal activation of all T cells after stimulation with anti-CD3.

In our studies, we observed a direct effect of trastuzumab on breast cancer cells MCF-7 *in vitro* and enhanced trastuzumab-mediated lysis in the presence of PBL which mediate ADCC. In addition, we demonstrated an enhanced lysis of HER2-expressing tumor cells by the combination of trastuzumab and PBL in PDAC cell lines established from primary cells (stage G1–G3, e.g., PancTu-I and Panc1) as well as in OC cells such as cisplatin-resistant SK-OV-3 cells and primary tumor cells OC1 and OC11.

Besides an initial existing resistance against anti-HER2 treatment, a further not completely understood problem seems to be an evolving secondary HER2-resistance. Several reasons are suggested as follows (6, 10, 49): (i) diminished HER2 expression, (ii) accumulation of an intracellular truncated active kinase p95-HER2 which lacks an extracellular domain (60, 61), (iii) overexpression of other EGFR family members, (iv) hindrance of trastuzumab binding, (v) hyper-activation of the downstream PI3K-Akt-mTOR pathway based on genetic alterations of the tumors (62), (vi) competing circulating immunoglobulins for FcγR (e.g., CD16) binding, (vii) cleavage of CD16 from the cell surface of NK cells by matrix metalloproteinases (63), (viii) undesired binding of inhibitory FcγR instead of activating ones, or (ix) loss of Th1 CD4^+^ T cells in the tumor microenvironment which supports cytotoxic T cells, such as CD8^+^ αβ TCR^+^ or γδ TCR^+^ cells (5, 64, 65).

Novel strategies are in development aiming to achieve more effective and durable responses. An attempt to combat trastuzumab resistance was the development of the ADC trastuzumab emtansine (T-DM-1). The cytotoxic agent emtansine (DM-1), which destroys tumor cells by binding to tubulin, has been conjugated to trastuzumab which inhibits the PI3K signaling (13, 49). An improvement of progression-free and overall survival in heavily pretreated patients with advanced HER2^+^ breast cancer and an acceptable toxicity profile has been demonstrated in phase III clinical trials (66, 67). Ongoing trials evaluate the role of T-DM-1 in neoadjuvant treatment of HER2^+^ breast cancer (68). In parallel, the development of further ADC such as SYD985 which are tested in ongoing phase I clinical trials, is enforced (49).

Another possibility can be a Th1 cytokine-induced senescence in tumor cells (5, 64, 65). The treatment of HER2-expressing breast cancer cells with Th1 cytokines, IFN-γ and TNF-α can cause oncogene inactivation of HER2 followed by a cell-cycle arrest of the tumor cells (5, 64). Since IFN-γ/TNF-α treatment of patients is not advisable, HER2^+^ breast cancer patients were treated with HER2-pulsed dendritic cells (DC) to restore depressed Th1 response which seems to be due to a functional and reversible cytokine shift (69). Datta et al. reported that a preserved anti-HER2 Th1 response was associated with pCR to neoadjuvant trastzumab application combined with chemotherapy (70). In addition, the anti-HER2 DC vaccination was combined with anti-estrogen therapy which improved regional nodal immune response and pCR rate in patients with estrogen receptor^+^/HER2^+^ early breast cancer (71).

A very promising strategy to overcome certain limitations of mAb and above-mentioned HER2-resistance mechanisms may be the use of bsAb with an enhanced cytotoxic activity against tumor cells (24). Sahied et al. designed bispecific minibodies targeting HER2 and CD16 which possess the advantages of a native IgG with respect to flexibility but in a different format. These minibodies have the potential to target HER2-expressing tumor cells and promote their lysis by NK cells and mononuclear phagocytes. Here, we employed the tribody [(HER2)_2_xCD16] to examine whether γδ T cells within PBL or TIL of PDAC and OC patients can be activated additionally to NK cells. Besides their HLA-independent recognition of antigens and their reduced induction of a graft-*versus*-host disease, an additional advantage of simultaneously activated γδ T cells is their capacity to present antigens, their possibility to induce maturation of DC, and their production of Th1 cytokines such as IFN-γ and TNF-α, which could compensate for a decreased number of CD4^+^ T cells (producing Th1 cytokines) at the tumor-site of PDAC patients (33–35, 72). Regarding the role of γδ T cells in the uptake of antigens *via* Ab-opsonization and CD16 and their possibility to cross-present antigens to αβ T cells after their activation (33, 73), we can speculate that tumor antigens could be taken up by γδ T cells and presented to αβ T cells after activation based on our observation that PAg and tribody [(HER2)_2_xCD16] stimulation induce a γδ T-APC phenotype. In contrast to the activation of NK cells by [(HER2)_2_xCD16], our results revealed that γδ T cells have to be concomitantly activated by their selective antigens and IL-2 in addition to the tribody to exert enhanced cytotoxic activity. Regarding a pre-activation of γδ T cells, an initial application of clinically licensed n-BP or PAg together with IL-2 is necessary for γδ T cells expansion to guarantee their enhanced cytotoxic activity by tribody [(HER2)_2_xCD16].

Our results clearly demonstrate that γδ T cells within PBL or TIL can be activated *via* a combined stimulation by their selective antigens plus IL-2 plus tribody [(HER2)_2_xCD16] to effectively lyse allogeneic as well as autologous tumor cells *via* the release of granzyme B. In general, cytotoxic γδ T cells often release granzymes out of their cytolytic granules after their activation by bsAb, which could be an advantage regarding the aspect that several tumor cells are almost resistant to the CD95- or TRAILR-induced cell death (38, 47). In addition, an adoptive transfer of γδ T cells with [(HER2)_2_xCD16] or [(HER2)_2_xVγ9] could enhance their cytotoxicity and support the anti-tumor response of innate cells activated *via* [(HER2)_2_xCD16]. Regarding the reported anti-tumor response of macrophages and neutrophils, an additional activation of γδ T cells at the tumor-site can be promising with respect to the differential infiltration of innate cells and γδ T cells in different tumor entities (74). If a bsAb targeting two HER2 molecules should be replaced by a bsAb targeting HER2/HER3 or HER2/HER4 or other families of the EGFR family has to be evaluated but the cognate activation of innate and innate-like cells (e.g., γδ T cells) *via* CD16 or other common ligands such as NKG2D-specific ligands for NK cells and γδ T cells seems to be promising (75).

## Ethics Statement

Patient cohorts leukocyte concentrates from healthy adult blood donors were obtained from the Department of Transfusion Medicine of the University Hospital Schleswig-Holstein (UKSH) in Kiel, Germany. Heparinized blood was drawn from HDs of the Institute of Immunology [UKSH, Christian-Albrechts University (CAU)], whereas blood from patients was obtained from the Department of General and Thoracic Surgery or the Clinic of Gynecology and Obstetrics, both of the UKSH in Kiel. In accordance with the Declaration of Helsinki, written informed consent was obtained from all donors, and the research was approved by the relevant institutional review boards (ethic committee of the Medical Faculty of the CAU to Kiel, code number: D405/10, D403/14, D404/14, AZ A157/11, AZ B3277/10).

## Author Contributions

HO, CK, MP, and DW performed the conception and the experimental design. The experiments, the analysis, and interpretation of the data and the preparation of the figures were done by HO, CK, MP, DG, and DW. SS and DB organized and provided the blood and tissue from PDAC and ovarian cancer patients, respectively. DK and MG provided the infrastrcuture for experimental set up and were helpful with the interpretation of the results. DW and HO wrote the manuscript. CK, MP, DK, SS, DB, DG, and MG revised the manuscript critically. All authors read and approved the final version of the manuscript.

## Conflict of Interest Statement

The authors declare that the research was conducted in the absence of any commercial or financial relationships that could be construed as a potential conflict of interest. The reviewer EL and handling Editor declared their shared affiliation.

## Supplementary Material

Figure S1 Impact of trastuzumab on cancer cells with different human epidermal growth factor receptor 2 (HER2) expression. **(A)** 5 × 10^3^ Panc1 cells, 5 × 10^3^ SK-OV-3 cells and 5 × 10^3^ primary ovarian cancer OC11 [(i), green lines] had been cultured in 10 % FCS medium for 26 h in plates which measured the impedance of the adherent tumor cells with electronic sensors. Impedance is expressed as an arbitrary unit called cell index (CI) every 5 min, which was normalized to 1 shortly before the addition of substances as follows: 10 µg/mL trastuzumab [(iii), brown line], PBL of healthy donors (E/T ratio 50:1) together with 50 IU/mL rIL-2 [(iv), purple line], or a combination of (iii) and (iv) as indicated [(v), blue line]. Triton-X-100 was added as positive control to induce maximal lysis [(ii), black line, positive control]. CI was then measured every minute for analysis of precise cytotoxicity time point for additional 40 h. The trastuzumab and/ or PBL-mediated lysis of HER2-expressing tumor cells correlated with the loss of tumor cell impedance and thus a decrease of the normalized CI. The average of triplicates and SD are presented; one representative experiment out of three is shown. **(B)** HER2 expression of Panc1 cells, SK-OV-3 cells as well as primary ovarian cancer cells OC11 was analyzed by staining the cells with 10 µg/mL trastuzumab (grey histograms) and appropriate isotype controls (open black lines) as indicated, following by appropriate second step Ab and measuring by flow cytometry. Numbers indicate the median fluorescence intensity of the appropriate staining with trastuzumab.

Figure S2 Control constructs and tribody [(HER2)_2_xCD16] did not modulate impedance of tumor cells. 5 × 10^3^ pancreatic ductal adenocarcinoma cells (Panc89 and PancTu-I) were cultured with medium (green line), control constructs such as 1 µg/mL tribody [(HER2)_2_xCD89] (purple line) or [(CD20)_2_xCD16] (light blue line), respectively or 1 µg/mL [(HER2)_2_xCD16] (red line) or with Triton-X-100 (black line) for the indicated time points. The cell index (CI) was analyzed in 5 min steps over ~24 h and in 1 min steps after 24 h. The average of three replicates with SD is presented for each tumor cell line in independent experiments.

Figure S3 Analysis of CD16 expression on short-term activated γδ T cells. For flow cytometric analysis, a gate was set on expanded Vδ2 γδ T cells with a purity of >95% (based on forward and side scatter properties to exclude dead cells) and on pan T cell receptor γδ-positive cells to determine the relative percentage of CD16-expressing Vδ2 γδ-positive T cells from healthy donors (*n* = 11) and from pancreatic ductal adenocarcinoma patients (*n* = 11). Each symbol presents the data of one donor, and the thick bars represent the mean value of different experiments. Statistical analysis was performed by *t* test. Significances are shown as *P* value; **P* < 0.05.

Figure S4 γδ T cell cytotoxicity against human epidermal growth factor receptor 2 (HER2)-positive PancTu-I cells and HER2-negative Raji cells with tribody [(HER2)_2_xCD16]. Vγ9Vδ2 γδ T cell lines of three healthy donors were co-cultured with complete medium (no bispecific antibodies), 1 µg/mL control tribody [(CD20)_2_xCD16] or 1 µg/mL tribody [(HER2)_2_xCD16] in the presence or absence of 300 nM phosphoantigen bromohydrin-pyrophosphate as indicated. γδ T cell lines at the indicated E/T ratios were added to ^51^Cr-labeled HER2-positive PancTu-I cells or HER2-negative Raji cells for 4 h. Each bar represents the mean value of three independent donors in triplicate assays. Statistical analysis was performed by ANOVA. Significances are shown as *P* value; **P* < 0.05 and ***P* < 0.01.

Figure S5 Tribody [(HER2)_2_xCD16] enhanced lysis of human epidermal growth factor receptor 2 (HER2)-expressing cancer cells in comparison to trastuzumab. 5 × 10^3^ Panc89 cells or esophageal cell line OE33 were cultured overnight in complete medium. After reaching the linear growth phase, Vγ9Vδ2 T cell lines at the indicated E/T ratio and substances were added as follows: without effector cells: complete medium (green line) or Triton-X-100 (black line); with effector cells and 12.5 IU/mL rIL-2 in medium (orange line), with 10 µg/mL trastuzumab (blue line), or with 1 µg/mL tribody [(HER2)_2_xCD16] (red line). The cell index was analyzed in 5 min steps over ~28–53 h and after normalization to 1 (after addition of PBL ± substances) in 1 min steps for >10 h. The average of three replicates with SD is represented for each tumor cell line with effector cells of one representative healthy donor (HD) in independent experiments (left panel). For validation, experiments were replicated several times under equal conditions using different Vγ9Vδ2 T cell lines of different donors in independent experiments (right panel). The cytotoxic capacity of Vγ9Vδ2 T cell lines against the indicated tumor cells calculated 4 h after addition of effector cells as % of specific lysis compared to control sample (green line) and maximal lysis (black line). Statistical analysis was performed by *t* test. Significances are shown as *P* value; ***P* < 0.01.

## Abbreviations

BrHPP, bromohydrin-pyrophosphate; bsAb, bispecific antibodies; HD, healthy donors; HER2, human epidermal growth factor receptor 2; mAb, monoclonal antibodies; n-BP, nitrogen-containing bisphosphonates; OC, ovarian cancer; PAg, phosphorylated antigen; PBL, peripheral blood lymphocytes; pCR, pathological complete response; PDAC, pancreatic ductal adenocarcinoma; RTCA, real-time cell analyzer; TCR, T cell receptor; TIL, tumor-infiltrating lymphocytes.
