# Supplementary figures and images for: Tribody [(HER2)2xCD16] Is More Effective Than Trastuzumab in Enhancing γδ T Cell and Natural Killer Cell Cytotoxicity Against HER2-Expressing Cancer Cells
Source: Front Immunol. 2018 Apr 19;9:814. doi: 10.3389/fimmu.2018.00814 (PMC5916959; doi:10.3389/fimmu.2018.00814)

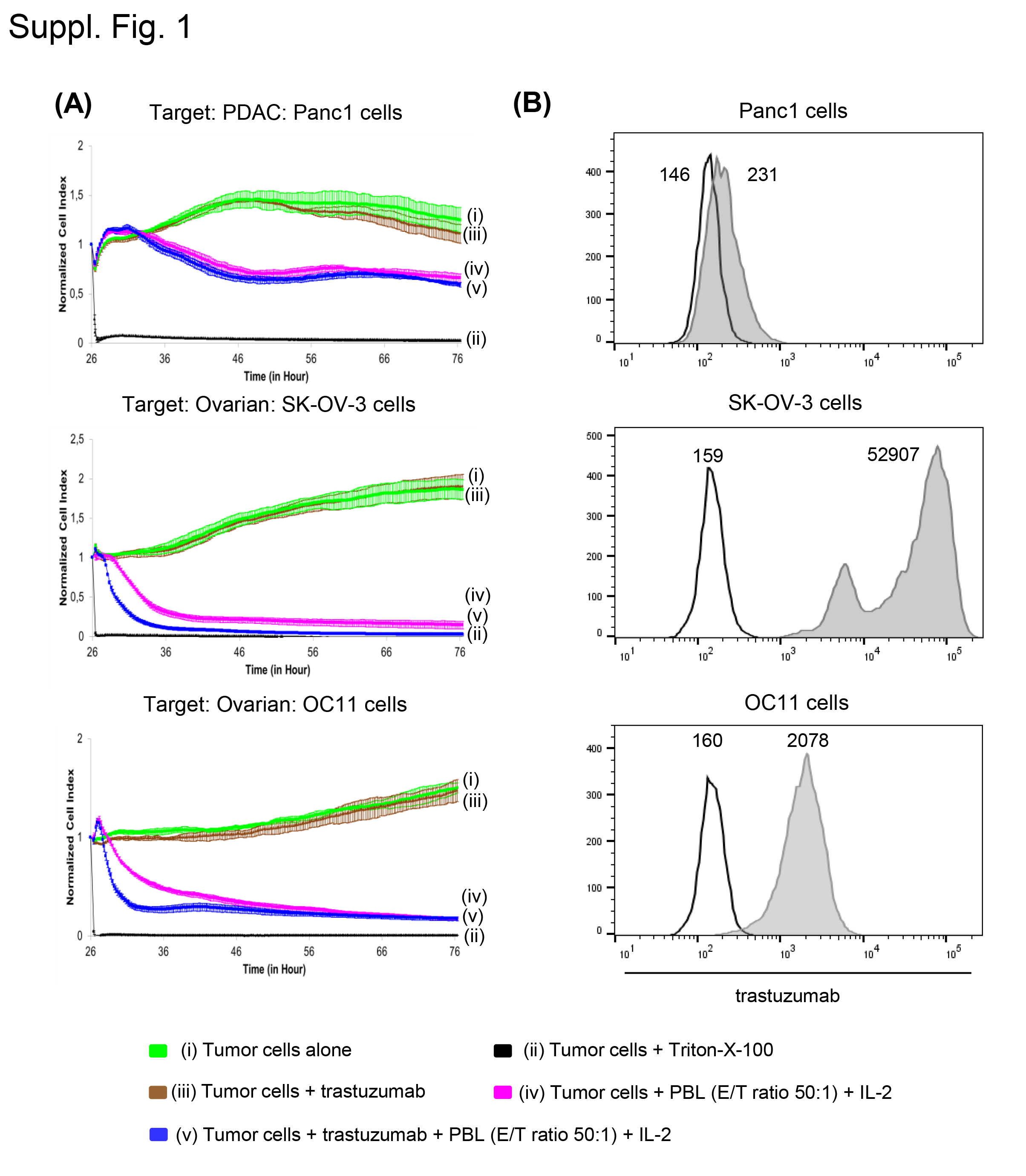

Supplement: Figure S1 — Impact of trastuzumab on cancer cells with different human epidermal growth factor receptor 2 (HER2) expression. (A) 5 × 10^3^ Panc1 cells, 5 × 10^3^ SK-OV-3 cells and 5 × 10^3^ primary ovarian cancer OC11 [(i), green lines] had been cultured in 10 % FCS medium for 26 h in plates which measured the impedance of the adherent tumor cells with electronic sensors. Impedance is expressed as an arbitrary unit called cell index (CI) every 5 min, which was normalized to 1 shortly before the addition of substances as follows: 10 µg/mL trastuzumab [(iii), brown line], PBL of healthy donors (E/T ratio 50:1) together with 50 IU/mL rIL-2 [(iv), purple line], or a combination of (iii) and (iv) as indicated [(v), blue line]. Triton-X-100 was added as positive control to induce maximal lysis [(ii), black line, positive control]. CI was then measured every minute for analysis of precise cytotoxicity time point for additional 40 h. The trastuzumab and/ or PBL-mediated lysis of HER2-expressing tumor cells correlated with the loss of tumor cell impedance and thus a decrease of the normalized CI. The average of triplicates and SD are presented; one representative experiment out of three is shown. (B) HER2 expression of Panc1 cells, SK-OV-3 cells as well as primary ovarian cancer cells OC11 was analyzed by staining the cells with 10 µg/mL trastuzumab (grey histograms) and appropriate isotype controls (open black lines) as indicated, following by appropriate second step Ab and measuring by flow cytometry. Numbers indicate the median fluorescence intensity of the appropriate staining with trastuzumab. [file Image_1.tif]

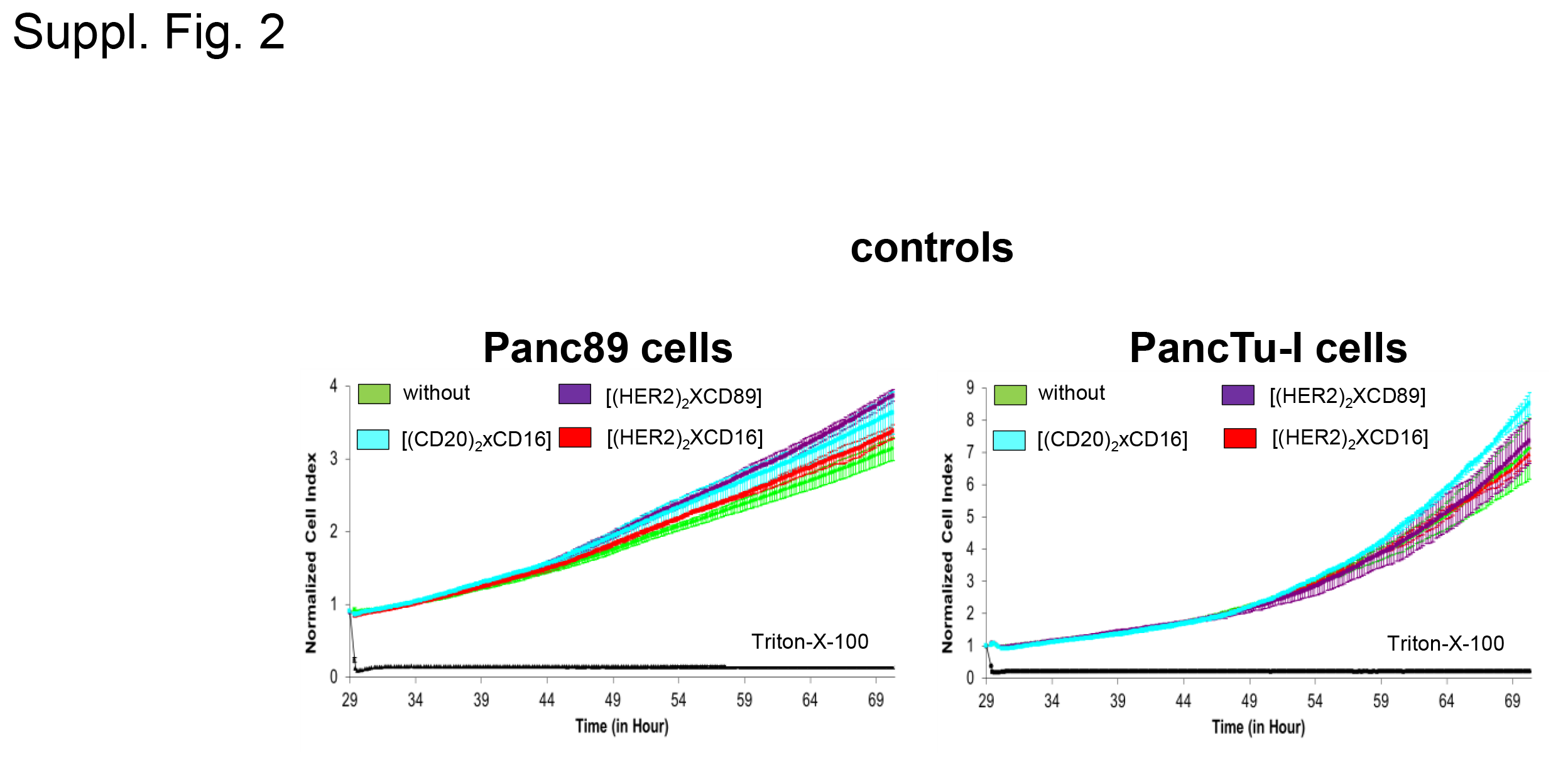

Supplement: Figure S2 — Control constructs and tribody [(HER2)2xCD16] did not modulate impedance of tumor cells. 5 × 10^3^ pancreatic ductal adenocarcinoma cells (Panc89 and PancTu-I) were cultured with medium (green line), control constructs such as 1 µg/mL tribody [(HER2)2xCD89] (purple line) or [(CD20)2xCD16] (light blue line), respectively or 1 µg/mL [(HER2)2xCD16] (red line) or with Triton-X-100 (black line) for the indicated time points. The cell index (CI) was analyzed in 5 min steps over ~24 h and in 1 min steps after 24 h. The average of three replicates with SD is presented for each tumor cell line in independent experiments. [file Image_2.tif]

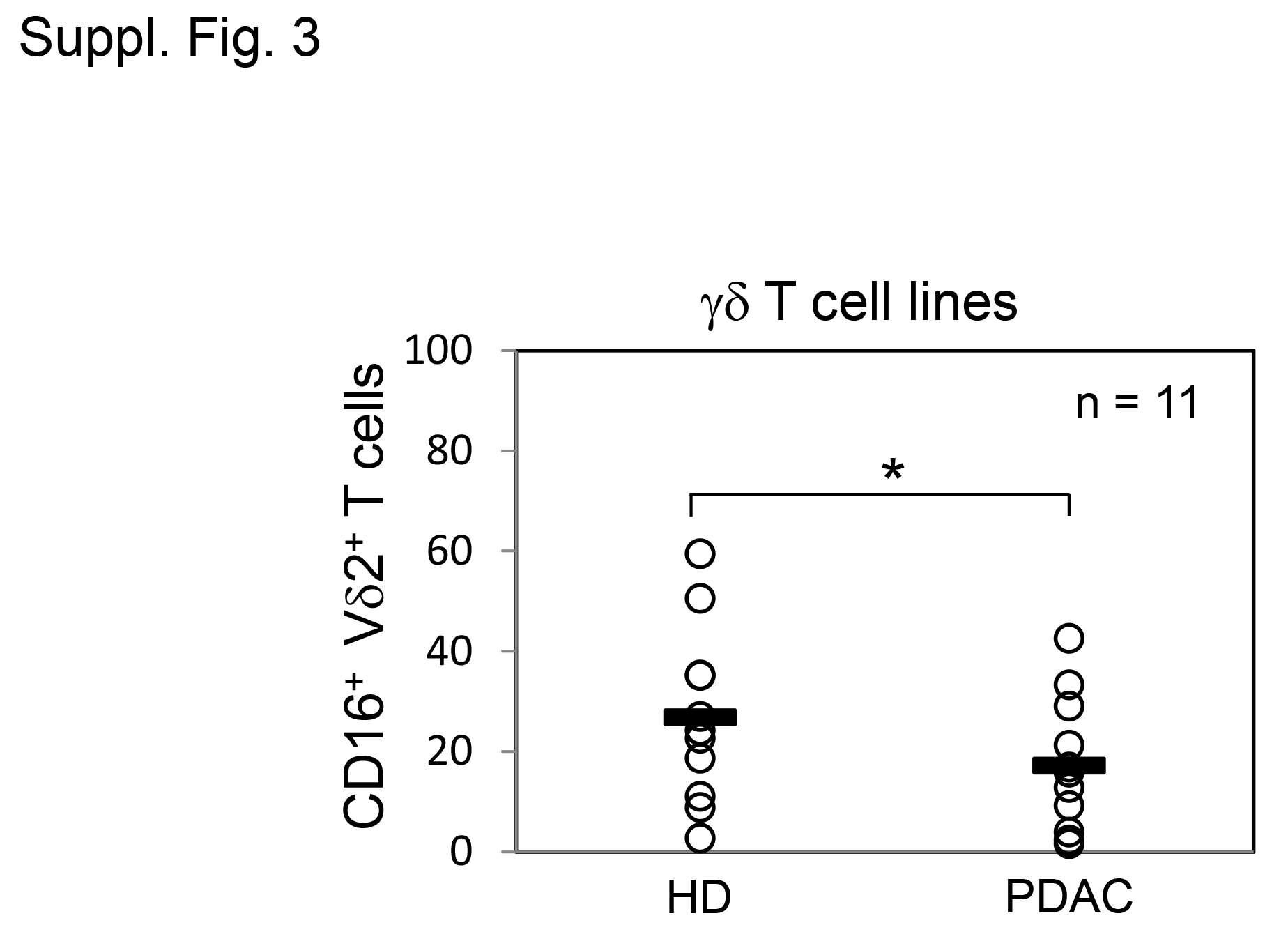

Supplement: Figure S3 — Analysis of CD16 expression on short-term activated γδ T cells. For flow cytometric analysis, a gate was set on expanded Vδ2 γδ T cells with a purity of >95% (based on forward and side scatter properties to exclude dead cells) and on pan T cell receptor γδ-positive cells to determine the relative percentage of CD16-expressing Vδ2 γδ-positive T cells from healthy donors (n = 11) and from pancreatic ductal adenocarcinoma patients (n = 11). Each symbol presents the data of one donor, and the thick bars represent the mean value of different experiments. Statistical analysis was performed by t test. Significances are shown as P value; *P < 0.05. [file Image_3.tif]

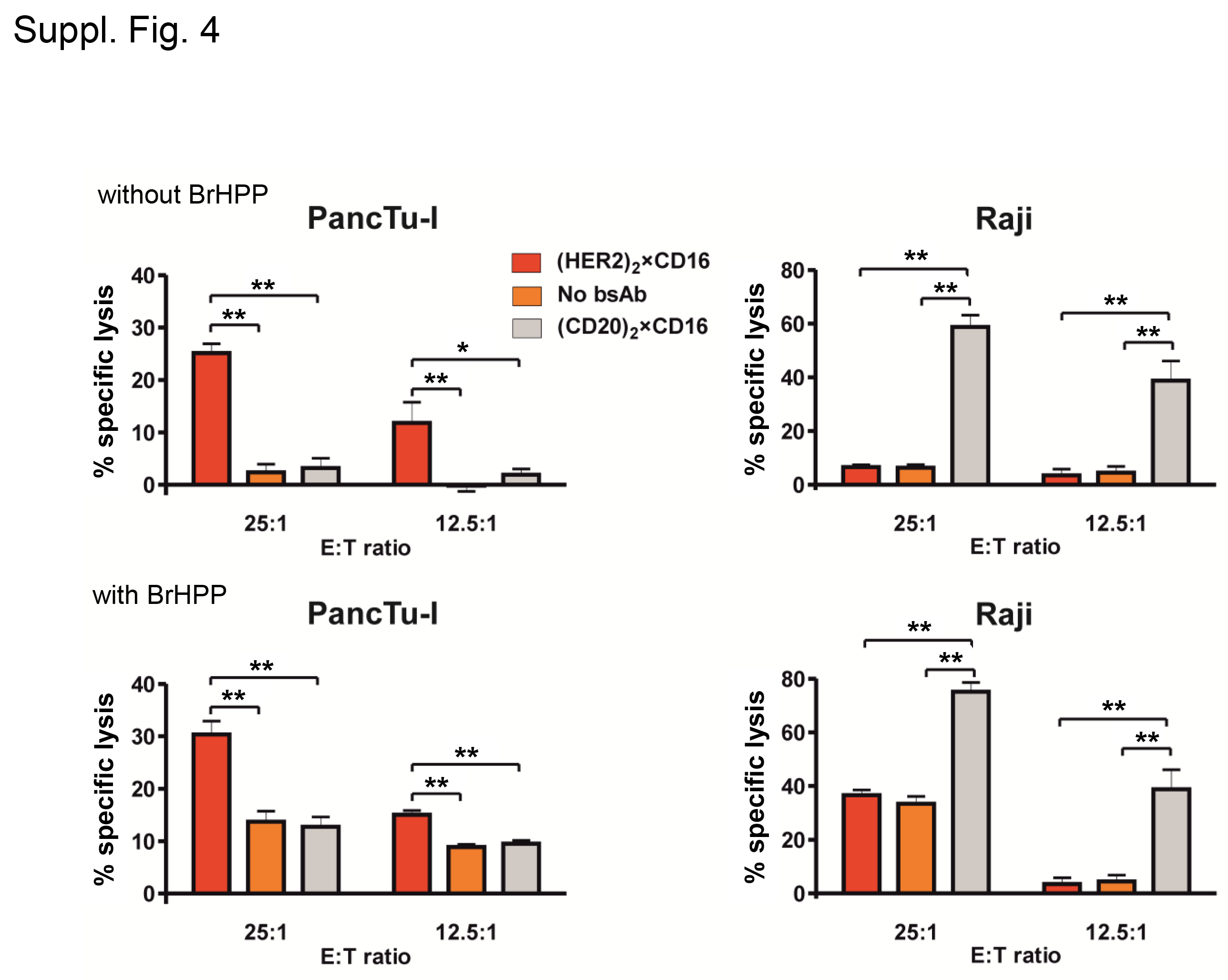

Supplement: Figure S4 — γδ T cell cytotoxicity against human epidermal growth factor receptor 2 (HER2)-positive PancTu-I cells and HER2-negative Raji cells with tribody [(HER2)2xCD16]. Vγ9Vδ2 γδ T cell lines of three healthy donors were co-cultured with complete medium (no bispecific antibodies), 1 µg/mL control tribody [(CD20)2xCD16] or 1 µg/mL tribody [(HER2)2xCD16] in the presence or absence of 300 nM phosphoantigen bromohydrin-pyrophosphate as indicated. γδ T cell lines at the indicated E/T ratios were added to 51Cr-labeled HER2-positive PancTu-I cells or HER2-negative Raji cells for 4 h. Each bar represents the mean value of three independent donors in triplicate assays. Statistical analysis was performed by ANOVA. Significances are shown as P value; *P < 0.05 and **P < 0.01. [file Image_4.tif]

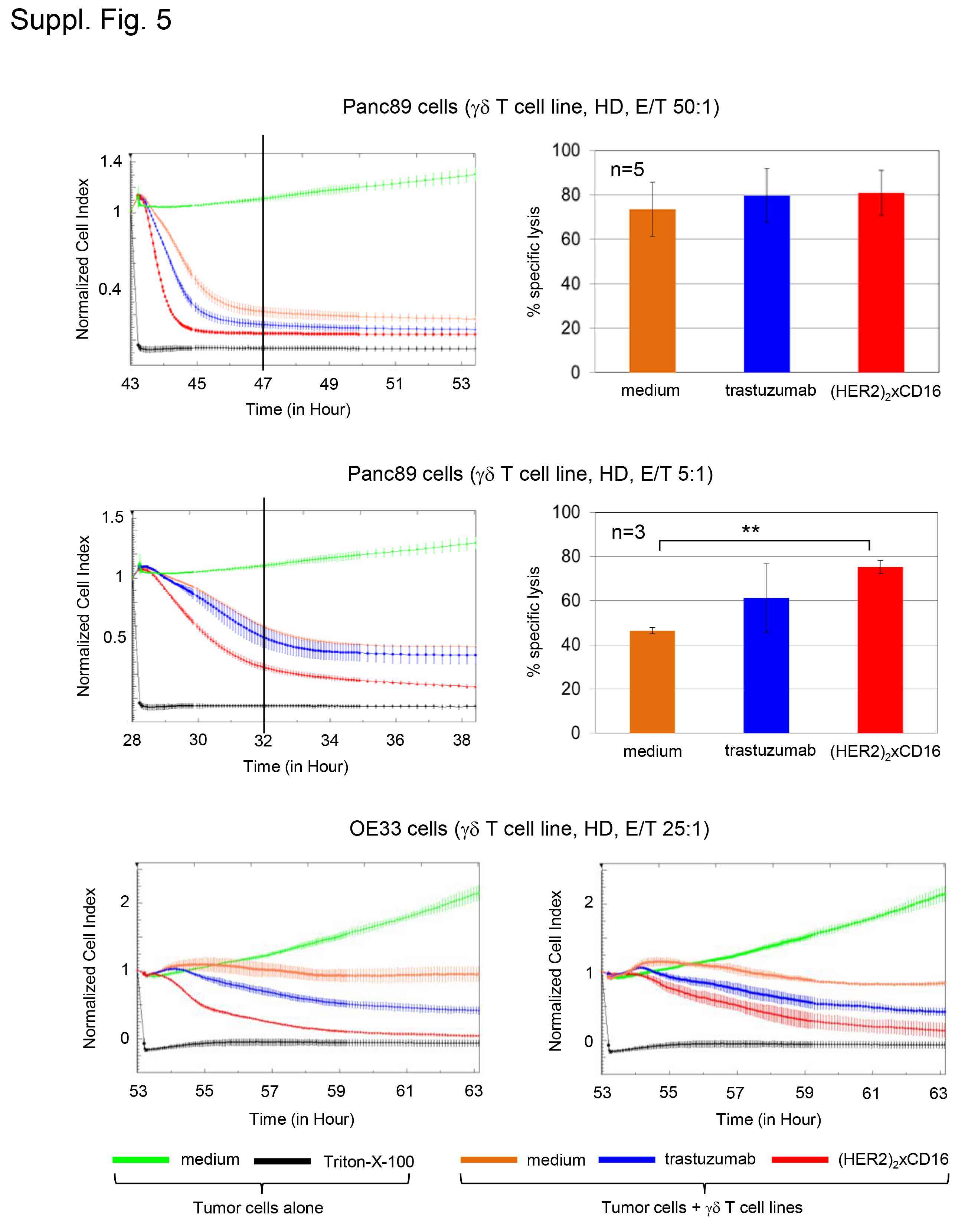

Supplement: Figure S5 — Tribody [(HER2)2xCD16] enhanced lysis of human epidermal growth factor receptor 2 (HER2)-expressing cancer cells in comparison to trastuzumab. 5 × 10^3^ Panc89 cells or esophageal cell line OE33 were cultured overnight in complete medium. After reaching the linear growth phase, Vγ9Vδ2 T cell lines at the indicated E/T ratio and substances were added as follows: without effector cells: complete medium (green line) or Triton-X-100 (black line); with effector cells and 12.5 IU/mL rIL-2 in medium (orange line), with 10 µg/mL trastuzumab (blue line), or with 1 µg/mL tribody [(HER2)2xCD16] (red line). The cell index was analyzed in 5 min steps over ~28–53 h and after normalization to 1 (after addition of PBL ± substances) in 1 min steps for >10 h. The average of three replicates with SD is represented for each tumor cell line with effector cells of one representative healthy donor (HD) in independent experiments (left panel). For validation, experiments were replicated several times under equal conditions using different Vγ9Vδ2 T cell lines of different donors in independent experiments (right panel). The cytotoxic capacity of Vγ9Vδ2 T cell lines against the indicated tumor cells calculated 4 h after addition of effector cells as % of specific lysis compared to control sample (green line) and maximal lysis (black line). Statistical analysis was performed by t test. Significances are shown as P value; **P < 0.01. [file Image_5.tif]
